# A multimodal fusion model integrating Vision Transformer, radiomics, and clinical features for predicting bone metastasis in prostate cancer

**DOI:** 10.3389/fonc.2026.1841761

**Published:** 2026-07-06

**Authors:** Guobo Li, Liqiu Liu, Zuliang Xu, Zhenmei Huang, Tao Zheng, Zishan Liu, Guoyu Wang, Dabin Ren

**Affiliations:** 1 Department of Radiology, Taizhou Central Hospital (Taizhou University Hospital), Taizhou, Zhejiang, China; 2 Clinical Medical College, Jiamusi University, Jiamusi, Heilongjiang, China

**Keywords:** bone metastasis, fusion model, prostate cancer, radiomics, Vision Transformer

## Abstract

**Objectives:**

To evaluate the performance of a multimodal fusion framework integrating a Vision Transformer (ViT), radiomics, and clinical features for predicting bone metastasis (BM) status in patients with prostate cancer (PCa).

**Methods:**

Patients with pathologically confirmed PCa were retrospectively included. Based on clinical features and apparent diffusion coefficient (ADC) images, three single-modal models were constructed: the clinical model (Model_Clin), the radiomics model (Model_Rad), and the ViT model (Model_ViT). Subsequently, a multimodal fusion model (Model_Fusion) was constructed by integrating ViT, radiomics, and clinical features. Model performance was evaluated using the receiver operating characteristic (ROC) curve and the DeLong test. The clinical utility and interpretability of the Model_Fusion were assessed using decision curve analysis (DCA) and Shapley additive explanations (SHAP).

**Results:**

Model_ViT demonstrated the best performance among the single-modal models, achieving AUCs of 0.909 and 0.872 in the training and validation sets, respectively, outperforming both Model_Rad (AUC = 0.885 and 0.842) and Model_Clin (AUC = 0.861 and 0.781). By integrating multimodal information, Model_Fusion achieved superior performance (AUC = 0.944 and 0.894). DeLong test results showed that, in the training set, Model_Fusion had a significantly higher AUC than Model_Clin, Model_Rad, and Model_ViT (all *P* < 0.05), whereas in the validation set, a significant difference was observed only when compared with Model_Clin. DCA further demonstrated that Model_Fusion provided a higher net benefit. SHAP analysis indicated that the predicted probability of ViT contributed the most to Model_Fusion.

**Conclusion:**

A fusion model integrating ViT, radiomics, and clinical features provides a non-invasive framework for predicting BM in PCa, which may help guide personalized clinical decision-making and prognostic evaluation.

## 1 Introduction

Prostate cancer (PCa) ranks as the second most commonly diagnosed malignancy and the fifth leading contributor to cancer-related mortality in men globally, with an increasing incidence globally (1, 2). Bone metastasis (BM) represents the most common form of distant metastasis in PCa, accounting for more than 80% of metastatic sites. Patients with BM exhibit significantly poorer prognoses and higher mortality rates than those without BM. However, the majority of patients with BM remain asymptomatic at an early stage, and effective early diagnostic approaches are lacking (3, 4). Moreover, patients with BM are generally no longer candidates for surgical intervention, and their management relies on palliative care, androgen deprivation therapy, chemotherapy, and radiotherapy (5). Therefore, accurate prediction of BM status in PCa patients is critical for personalized clinical decision-making and prognostic assessment.

Previous research has confirmed that clinical symptoms and prostate-specific antigen (PSA) levels are not reliable biomarkers for identifying BM in PCa patients (6). Although computed tomography (CT) offers certain advantages in detecting bone lesions and evaluating bone destruction, it has limitations in distinguishing small osteoblastic metastases from bone islands, in addition to limitations in assessing metastatic activity and treatment response. Although bone scintigraphy is widely applied in clinical practice, it is associated with radiation exposure and high medical costs (7). Magnetic resonance imaging (MRI) exhibits high sensitivity and specificity for BM detection. However, its diagnostic performance may be compromised by heterogeneous imaging manifestations, which may lead to misinterpretation. Additionally, current imaging modalities may underestimate the actual incidence of BM (8). Therefore, the development of more advanced methods for predicting BM status is of great importance.

Radiomics, which converts medical images into high-dimensional, mineable data, has demonstrated substantial value in tumor diagnosis and prognostic assessment (9). Previous studies have shown that radiomics holds great promise for predicting biological behavior, evaluating therapeutic response, and estimating prognosis across a variety of malignancies, including lung, breast, colorectal, and prostate cancers (10–13). Furthermore, radiomics enables the characterization of tumor heterogeneity and the tumor microenvironment, thereby supporting precision medicine. Deep learning, as a key component of artificial intelligence, has markedly advanced image analysis by automating feature extraction and classification, thereby improving diagnostic efficiency and accuracy. The Vision Transformer (ViT), an emerging deep learning model, has demonstrated unique advantages in medical image analysis (14). By partitioning images into patches and modeling them as sequential inputs, ViT leverages self-attention mechanisms to capture long-range dependencies, enabling a deeper understanding of image content. In medical imaging, ViT has achieved promising performance in tasks such as tumor segmentation and classification (15, 16).

At present, there are no studies using ViT to predict BM status in PCa. This study aims to overcome the limitations of existing methods. Based on apparent diffusion coefficient (ADC) images, this study extracts robust high-dimensional radiomics features and introduces ViT to capture complex spatial structural information and potential patterns. A multimodal framework integrating ViT, radiomics, and clinical features was constructed to achieve comprehensive feature extraction, which was used to predict BM status in PCa, thereby providing support for personalized clinical management and treatment decision-making.

## 2 Materials and methods

### 2.1 Data collection

Ethical approval for this study was obtained from the Ethics Committee of Taizhou Central Hospital (Approval No. 2025L-08-150). Patients with pathologically confirmed PCa who were diagnosed between June 2018 and December 2025 were retrospectively included. The inclusion criteria were as follows: (I) MRI was performed using a 3.0 T scanner, and image quality met diagnostic requirements; (II) the lesion was clearly visible on MRI images, and its size was at least 5 mm; (III) ultrasound-guided biopsy or radical resection was performed within 2 weeks after MRI examination, and the Gleason score (GS) was obtained; and (IV) bone scintigraphy results were available. The exclusion criteria were as follows: (I) ultrasound-guided biopsy, surgery, or other treatments were performed before MRI examination; (II) poor image quality that could not be evaluated; (III) incomplete clinical data; (IV) the presence of other primary malignant tumors; and (V) a history of other bone injuries or bone metabolic disorders. To ensure comparability among different models and avoid bias and information leakage risks caused by data partitioning, this study adopted a unified data partitioning strategy. Stratified sampling based on the outcome variable was performed to divide the dataset into training and validation sets at a ratio of 7:3.

In this study, the diagnosis of BM was based on a comprehensive assessment of imaging findings, clinical data, and follow-up information. All patients underwent bone scintigraphy after the diagnosis of PCa. For cases with positive findings and high clinical suspicion of BM, CT or MRI examinations performed within one month were reviewed, and the diagnosis was confirmed through multidisciplinary discussion. For equivocal cases, follow-up imaging and clinical evaluation at 3 months were used for final determination. Patients with inconclusive results were excluded.

### 2.2 MRI acquisition parameters

MRI data were acquired on a 3.0 T scanner (Skyra, Siemens Healthineers, Erlangen, Germany) equipped with an 18-channel body coil. Participants were scanned in the supine, head-first position, with sufficient bladder distension maintained before imaging. The scanning center line was aligned with the pubic symphysis. The scanning sequences and parameters were as follows: (I) Axial T2WI sequence: TE 89 ms, TR 2400 ms, excitation times 2, FOV 220 mm × 220 mm, matrix 256 × 256, slice thickness 3 mm, flip angle 160°, and oversampling 80%. (II) Axial DWI replicated the T2WI sequence for scanning the positioning line: TR 4200 ms, TE 72 ms, excitation times 1, FOV 220 mm × 220 mm, matrix 256 × 256, slice thickness 3 mm, oversampling 50%, and b = 1000 s/mm^2^. The ADC map was automatically generated based on DWI.

### 2.3 Clinical feature analysis and model construction

Clinical data, including age, PSA, and GS, were collected. Prostate dimensions, including cranio-caudal diameter (CCD), transverse diameter (TD), and anterior–posterior diameter (APD), were measured on MR images, from which prostate volume (PV) and prostate-specific antigen density (PSAD) were calculated. Univariate logistic regression was initially conducted to evaluate the association between these variables and BM, and variables with *P* < 0.05 were selected. Multivariate logistic regression was subsequently performed using these variables to identify independent predictors of BM. Finally, the selected variables were used to construct a clinical model (Model_Clin) based on logistic regression.

The formula used to calculate PV was as follows: PV = 0.52 × TD × CCD × APD.

### 2.4 Image preprocessing and ROI segmentation

Before drawing the areas of interest (ROI), all images underwent standardized preprocessing to reduce differences in images collected by different MR scanners, improve overall image quality, reduce the interference of noise on subsequent segmentation, and improve segmentation accuracy. The preprocessing process included N4 bias field correction, Z-score standardization, and resampling to 1 × 1 × 1 mm^3^ voxels. Finally, the ADC images were imported into ITK-SNAP (http://www.nitrc.org/projects/itk-snap/), and ROIs were manually delineated slice by slice by two radiologists with 5 and 10 years of experience in abdominal imaging. Intra- and inter-observer agreement were assessed using the intraclass correlation coefficient (ICC), and an ICC value ≥ 0.80 was regarded as good agreement.

### 2.5 Radiomics model construction

**Figure 1:**
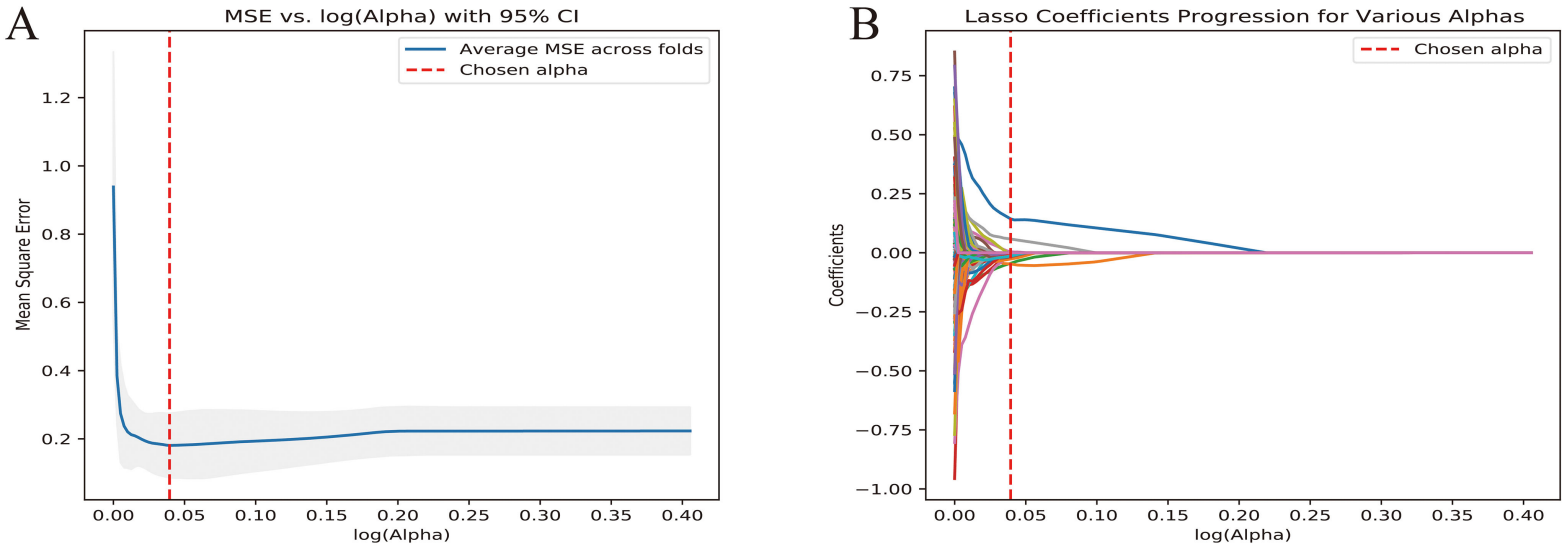
**(A)** shows the coefficients obtained using LASSO with five-fold cross-validation. **(B)** shows the cross-validated mean squared error as a function of the regularization parameter (λ) for radiomics features.

Radiomics features were extracted using PyRadiomics (https://github.com/radiomics/pyradiomics), and the full extraction protocol is provided in the **Supplementary Materials**. For feature selection and dimensionality reduction, all features were first standardized using Z-score normalization. We then assessed feature reproducibility using the ICC, retaining only features with ICC ≥ 0.80. Zero-variance features were removed using the variance threshold method, and the remaining features were screened using the t-test or Mann-Whitney U test, retaining only statistically significant features (*P* < 0.05). To reduce collinearity, Spearman correlation analysis was conducted, and features with r ≥ 0.8 were excluded. Finally, least absolute shrinkage and selection operator (LASSO) regression was used for further feature selection, with the optimal λ determined by five-fold cross-validation (**Figure 1**). The Rad-score was calculated from features with non-zero coefficients, and the calculation is detailed in the **Supplementary Materials**. For model construction, we combined grid search and five-fold cross-validation on the training set to optimize hyperparameters (**Supplementary Table 1**). A logistic regression model (Model_Rad) was then constructed using the Rad-score to predict BM status in PCa.

To avoid information leakage and ensure robust model evaluation, the entire radiomics workflow, including preprocessing, feature selection, and model construction, was performed exclusively in the training set. All parameters determined in the training set were then applied unchanged to the validation set. The Rad-score was calculated as a linear combination of selected features using coefficients derived from the training set and was directly applied to the validation set.

### 2.6 ViT model construction

**Figure 2:**
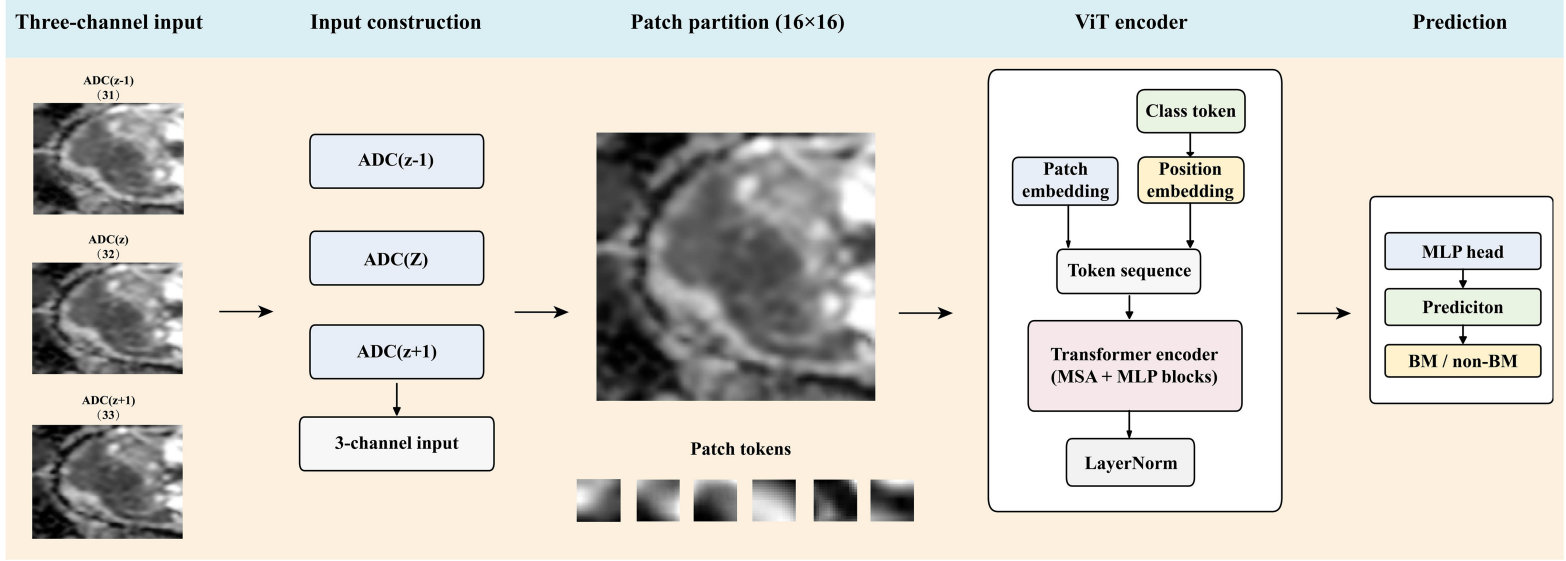
The slice with the largest lesion area is selected from ADC images as the central slice (z), and its adjacent slices (z−1 and z+1) are also extracted. The three slices are resized to a uniform size and combined to form a three-channel input. The input image is then divided into fixed-size patches (16 × 16). After patch embedding, the patches, together with the class token and positional embeddings, form a sequence of tokens, which is fed into the ViT encoder for feature learning. Finally, the prediction of BM is obtained through a MLP head. MLP, multi-layer perceptron; MSA, multi-head self-attention; BM, bone metastasis.

The imaging data, including ADC images and lesion ROI, were stored in NIfTI format. For each case, the data were first binarized, and then the lesion area was calculated slice by slice along the Z-axis. The slice with the largest tumor area was selected as the representative slice, and this slice, together with the adjacent slices above and below it, was extracted to form a three-channel input. All images were resized to 224 × 224 pixels, followed by pixel normalization and standardization. The dataset was divided based on the stratified sampling results of the radiomics model to ensure consistent class distributions between the training and validation sets. A random seed of 42 was used to enhance model stability and generalizability. The model adopted the ViT_small_patch16_224 network from the timm library as the backbone. The input image size was 224 × 224 pixels, and the patch size was 16 × 16 pixels. The input image was split into fixed-size patches and projected into feature embeddings. Through a multi-layer Transformer structure, the model used the self-attention mechanism to establish global dependencies between different image regions, thereby achieving deep representation learning of tumor imaging features. During model development, five-fold cross-validation was performed on the training set. In each fold, the model was trained on the training subset and evaluated on the validation subset, and the model with the highest validation AUC was selected. Early stopping was applied if the validation performance did not improve for five consecutive epochs. After model development, a final model was retrained on the full training set and evaluated on the internal validation set, which was not used at any stage of model development. Finally, Model_ViT for predicting BM was constructed based on the predicted probabilities (**Figure 2**). The training parameters of Model_ViT are detailed in the **Supplementary Materials**.

### 2.7 Fusion model construction

Based on Model_Clin, Model_Rad, and Model_ViT, a multimodal fusion model was constructed using logistic regression, with the predicted probabilities from each single-modal model as independent variables and BM status as the dependent variable. This integration of different information sources enabled the model to effectively leverage the complementary information among clinical features, radiomics features, and ViT deep features, thereby significantly enhancing the comprehensive discrimination ability of the model for BM in PCa. Moreover, logistic regression can adaptively learn the weights of the prediction results from each modality, which may partially mitigate the limitations of individual models and enhance model stability and generalizability.

### 2.8 Model evaluation

Receiver operating characteristic (ROC) curves were used to assess model performance, and the area under the curve (AUC), sensitivity, specificity, accuracy, positive predictive value (PPV), negative predictive value (NPV), and F1-score were calculated. The DeLong test was applied to assess differences in AUCs among different models. Calibration curves were constructed to assess the agreement between predicted probabilities and observed incidence, reflecting the calibration of the models. Decision curve analysis (DCA) was used to evaluate the clinical net benefit of the models. Furthermore, SHapley additive explanations (SHAP) were used to provide interpretability for the Model_Fusion. SHAP values were calculated for each feature to quantify its contribution to model predictions.

### 2.9 Statistical analysis

Statistical analyses were performed using SPSS software (www.ibm.com/products/spss-statistics). Continuous variables were expressed as mean ± standard deviation (SD), while categorical data were expressed as absolute counts and percentages. Group differences in continuous variables were assessed using t-tests for normally distributed data, whereas the Mann–Whitney U test was used for non-normally distributed data. Group differences in categorical variables were evaluated using the chi-square test. A *P* value < 0.05 was defined as a statistically significant difference.

## 3 Results

### 3.1 Clinical baseline characteristics

A total of 220 patients were randomly divided into training (n = 154) and validation (n = 66) sets at a 7:3 ratio using stratified sampling. There were 72 patients with BM and 148 patients without BM. **Table 1** shows that APD, CCD, PV, PSA, PSAD, and GS had statistically significant differences between the BM and non-BM groups (*P* < 0.05). In the training set, significant differences were observed between the two groups in APD, CCD, PV, PSA, PSAD, and GS (*P* < 0.05), as shown in **Table 2**. In the validation set, CCD, PV, PSA, PSAD, and GS showed statistically significant differences between groups (*P* < 0.05).

**Table 1:** Clinical characteristics of patients in the BM group and non-BM group.

Variables	Total (n = 220)	BM (n = 72)	Non-BM (n = 148)	*t/Z/χ^2^*	*P*
Age (years)	72.75 ± 8.30	74.01 ± 8.52	72.13 ± 8.15	-1.287	0.198
TD (cm)	4.89 ± 0.71	4.90 ± 0.79	4.88 ± 0.67	-0.060	0.952
APD (cm)	3.73 ± 0.87	4.00 ± 0.95	3.61 ± 0.80	-3.121	0.002
CCD (cm)	4.17 ± 1.00	4.48 ± 1.08	4.02 ± 0.93	-3.402	0.001^*^
PV(cm^3^)	43.17 ± 28.91	49.58 ± 29.31	40.05 ± 28.30	-2.909	0.004
PSA (ng/ml)	152.13 ± 288.04	345.32 ± 379.49	58.15 ± 163.93	-7.394	0.001^*^
PSAD (ng/ml/cm^3^)	3.93 ± 8.97	9.30 ± 13.77	1.32 ± 2.72	-6.837	0.001^*^
GS					
≤3 + 4	59 (26.8)	5 (6.9)	54 (36.5)	21.54	0.001^*^
≥4 + 3	161 (73.2)	67 (93.1)	94 (63.5)		

BM, bone metastasis; TD, transverse diameter; APD, anterior-posterior diameter; CCD, cranio-caudal diameter; PV, prostate volume; PSA, prostate-specific antigen; PSAD, prostate-specific antigen density; GS, Gleason score. *, *P* < 0.001.

**Table 2:** Clinical characteristics of patients in the training and validation sets.

Variables	Training set (n = 154)				Validation set (n = 66)			
	BM (n = 50)	Non-BM (n = 104)	t/Z/χ2	*P*	BM (n = 22)	Non-BM (n = 44)	t/Z/χ2	*P*
Age (years)	74.04 ± 8.45	72.47 ± 7.60	-0.885	0.376	73.95 ± 8.87	71.32 ± 9.37	-0.804	0.421
TD (cm)	4.89 ± 0.77	4.93 ± 0.74	-0.147	0.883	4.93 ± 0.85	4.76 ± 0.48	-0.355	0.723
APD (cm)	4.04 ± 0.97	3.66 ± 0.86	-2.574	0.010	3.91 ± 0.91	3.50 ± 0.63	-1.732	0.083
CCD (cm)	4.44 ± 1.00	4.13 ± 1.01	-2.263	0.024	4.56 ± 1.21	3.76 ± 0.66	-2.671	0.008
PV(cm^3^)	49.03 ± 25.65	42.78 ± 32.40	-2.087	0.037	50.83 ± 37.00	33.59 ± 12.71	-2.149	0.032
PSA (ng/ml)	392.21 ± 374.54	45.17 ± 108.83	-6.170	0.001^*^	349.85 ± 416.02	74.84 ± 168.42	-4.156	0.001^*^
PSAD (ng/mL/cm^3^)	9.85 ± 14.77	1.08 ± 2.17	-5.761	0.001^*^	8.04 ± 11.46	1.87 ± 3.67	-3.509	0.001^*^
GS								
≤3 + 4	3 (6.0)	36 (34.6)	14.620	0.001^*^	2 (9.1)	18 (40.9)	7.030	0.008
≥4 + 3	47 (94.0)	68 (65.4)			20 (90.9)	26 (59.1)		

BM, bone metastasis; TD, transverse diameter; APD, anterior-posterior diameter; CCD, cranio-caudal diameter; PV, prostate volume; PSA, prostate-specific antigen; PSAD, prostate-specific antigen density; GS, Gleason score. *, *P* < 0.001.

### 3.2 Clinical model performance

Univariate analysis revealed that, except for age and TD, all the other parameters were significantly associated with BM (*P* < 0.05). The subsequent multivariate analysis indicated that APD, CCD, PV, PSAD, and GS were independent predictors of BM, and the results are detailed in **Table 3**. Subsequently, multicollinearity was examined using variance inflation factor (VIF) analysis. The results showed that PV exhibited significant collinearity with other variables (VIF > 5) and was therefore excluded from the model. Finally, APD, CCD, PSAD, and GS were included to construct Model_Clin. The AUCs of Model_Clin were 0.861 (0.791–0.924) and 0.781 (0.661–0.884) in the training set and validation set, respectively.

**Table 3:** Univariate and multivariate analyses of clinical features.

Variables	Univariate analysis		Multivariate analysis	
	OR (95% CI)	*P*	OR (95% CI)	*P*
Age	1.029 (0.993–1.066)	0.115		
TD	1.043 (0.704–1.548)	0.832		
APD	1.681 (1.204–2.346)	0.002	2.142 (1.060–4.329)	0.034
CCD	1.595 (1.180–2.157)	0.002	2.742 (1.340–5.609)	0.006
PSA	1.004 (1.003–1.006)	0.001*		
PV	1.012 (1.001–1.023)	0.035	0.969 (0.941–0.998)	0.033
PSAD	1.258 (1.143–1.384)	0.001*	1.259 (1.047–1.516)	0.015
GS				
≤3 + 4		–		–
≥4 + 3	2.551 (1.258–5.174)	0.009	3.106 (1.261–7.649)	0.014

TD, transverse diameter; APD, anterior-posterior diameter; CCD, cranio-caudal diameter; PV, prostate volume; PSA, prostate-specific antigen; PSAD, prostate-specific antigen density; GS, Gleason score. *, *P* < 0.001.

### 3.3 Radiomics model performance

**Figure 3:**
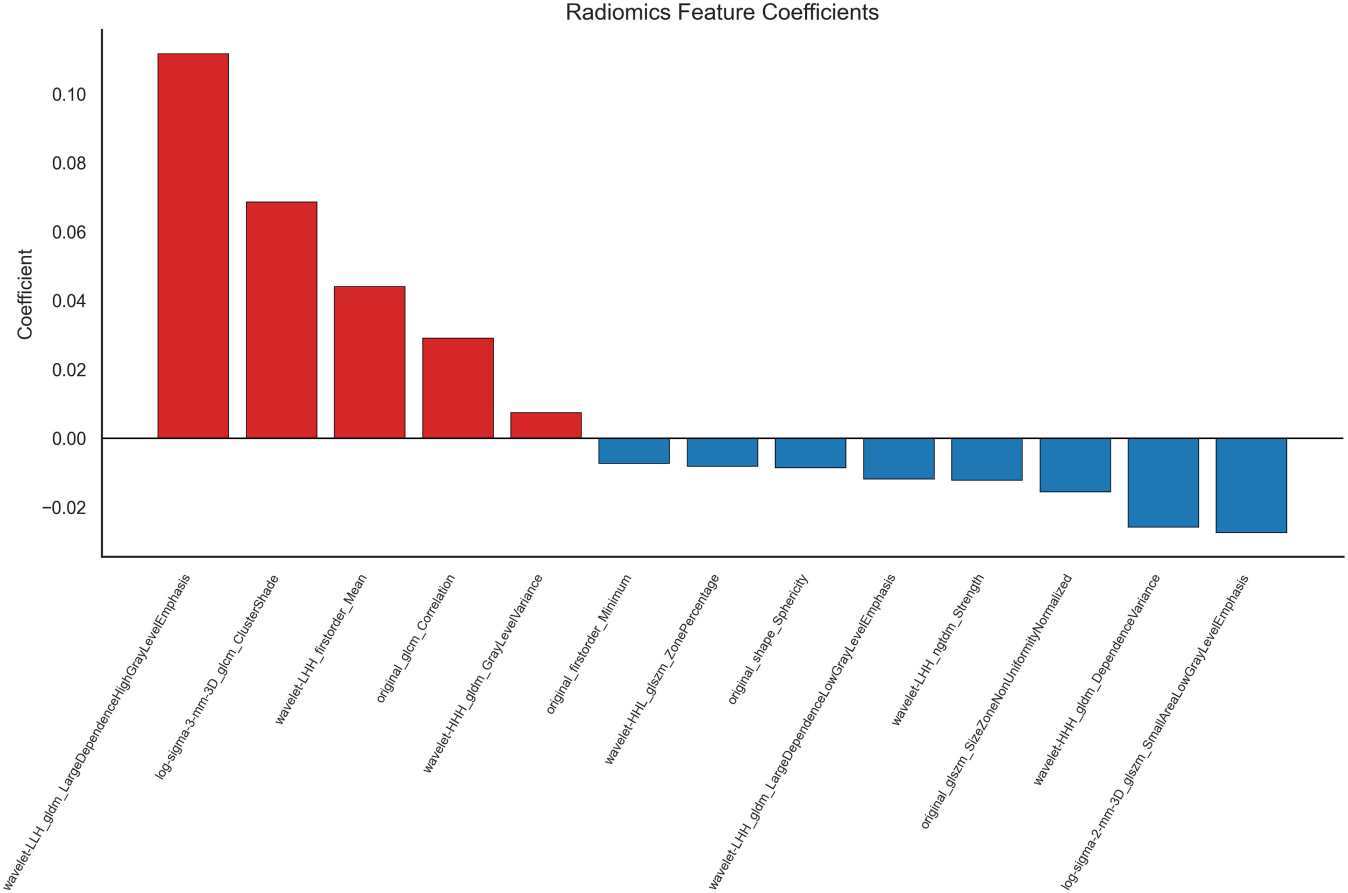
Selected radiomics features and their coefficients in the Model_Rad. The horizontal axis represents the selected features, and the vertical axis represents their corresponding coefficients. Red indicates positive coefficients, and blue indicates negative coefficients.

From each ROI, 1,130 radiomics features were extracted. After feature selection and dimension reduction, 13 optimal features were retained to construct Model_Rad. **Figure 3** shows the remaining optimal features and their weight coefficients after LASSO. The AUCs of Model_Rad were 0.885 (0.825–0.937) and 0.842 (0.736–0.928) in the training and validation sets, respectively, demonstrating the strong ability of the radiomics model to predict BM in PCa.

### 3.4 ViT model performance

The slice with the largest tumor area, along with its adjacent slices, was used to generate a three-channel input for the construction of Model_ViT. The AUC values of Model_ViT were 0.909 (0.855–0.955) and 0.872 (0.783–0.943) in the training and validation sets, respectively. The Model_ViT exhibited strong discriminative capability and generalization performance, outperforming Model_Rad and Model_Clin.

### 3.5 Fusion model performance

**Figure 4:**
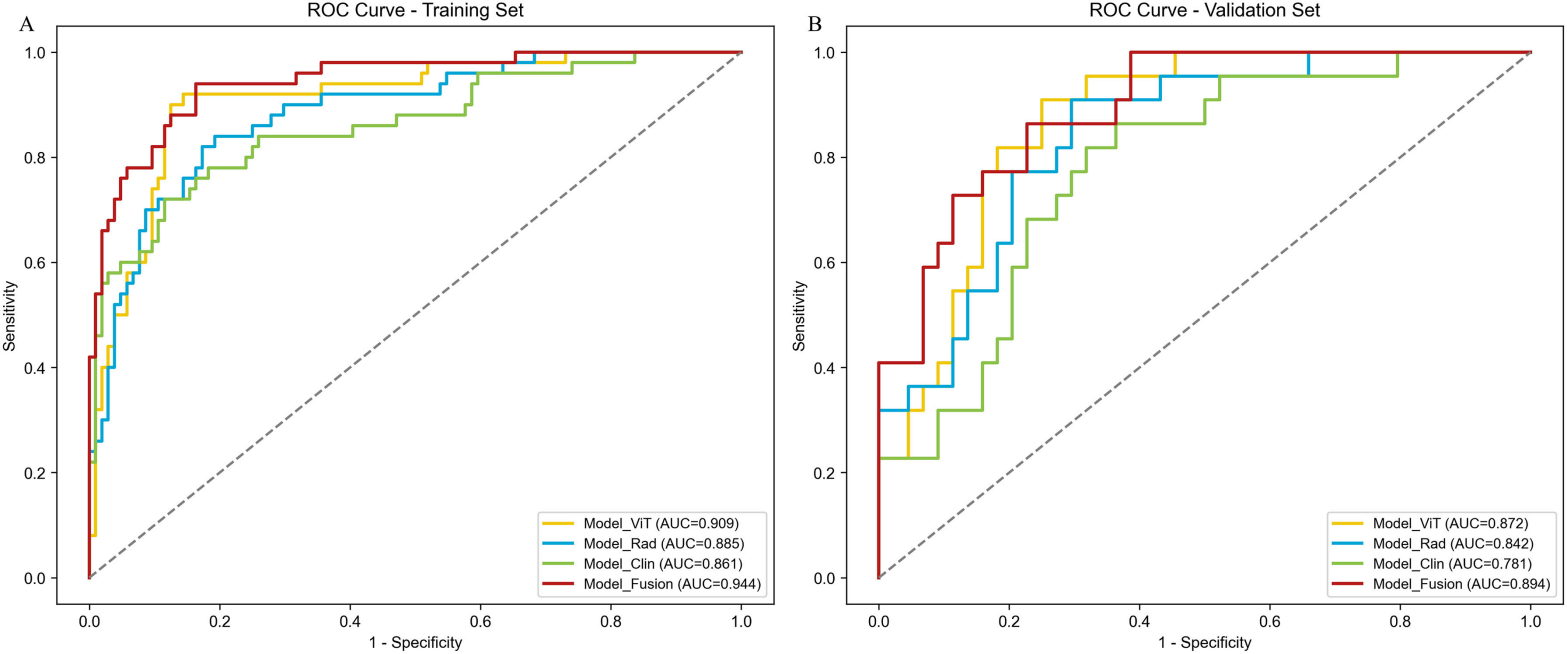
Receiver operating characteristic (ROC) curve analysis for predicting bone metastasis in prostate cancer. **(A)** The ROC curves in the training set; **(B)** The ROC curves in the validation set.

**Figure 5:**
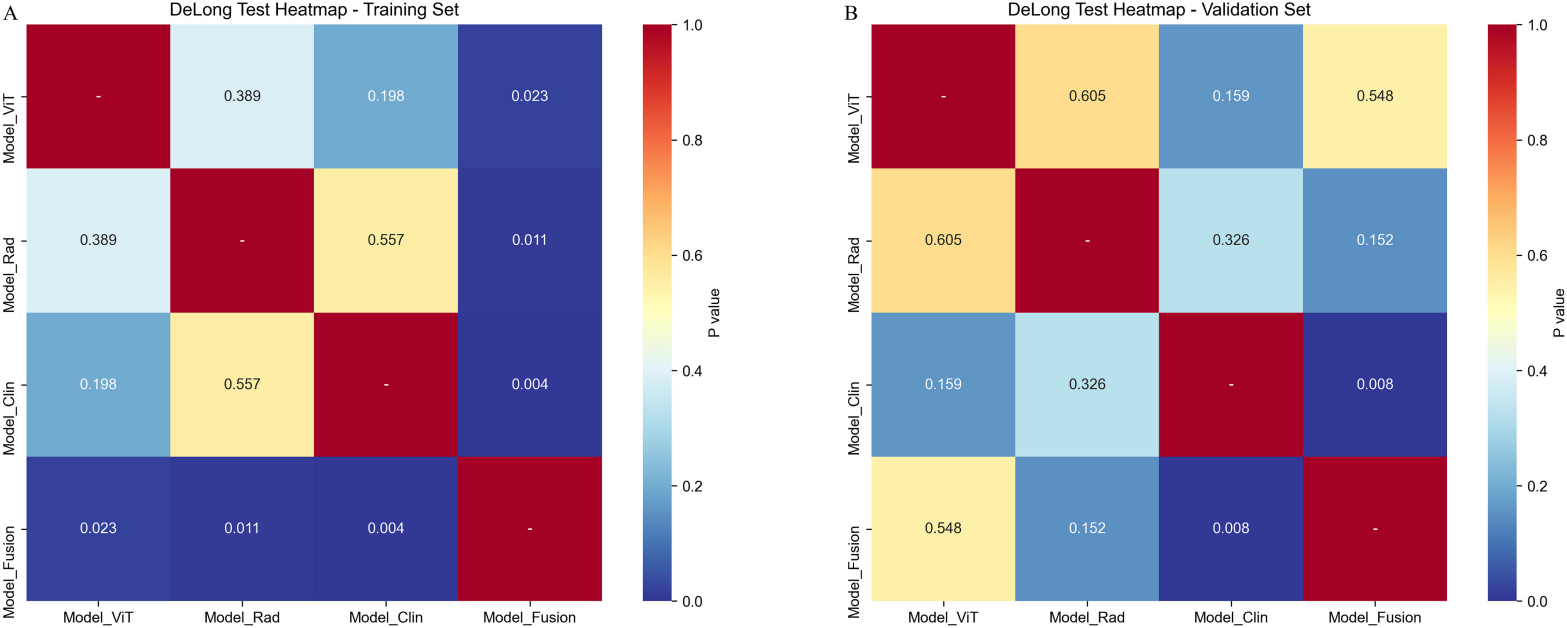
**(A)** Heatmap showing the *P*-values for AUC comparisons in the training set. **(B)** Heatmap showing the *P*-values for AUC comparisons in the validation set. A *P*-value < 0.05 indicates a statistically significant difference.

**Figure 6:**
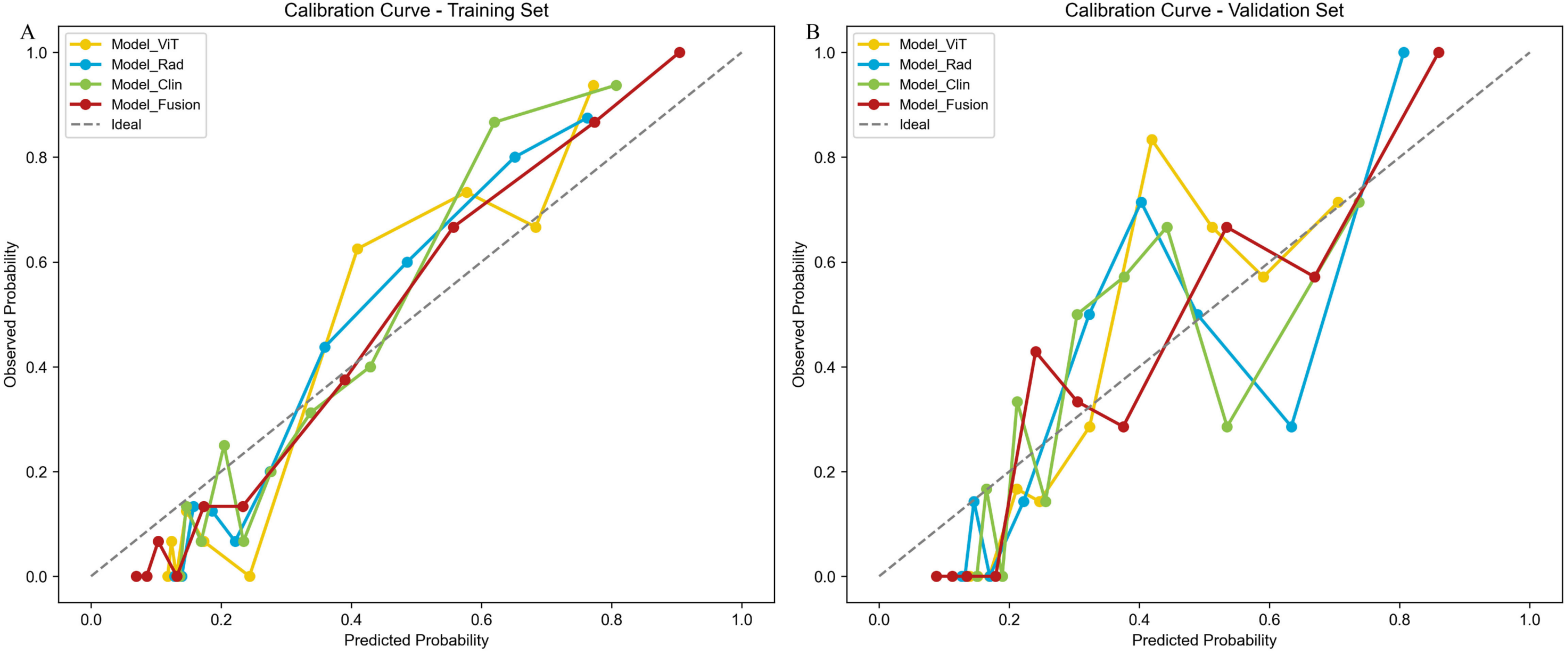
**(A)** The calibration curves in the training set; **(B)** The calibration curves in the validation set.

**Figure 7:**
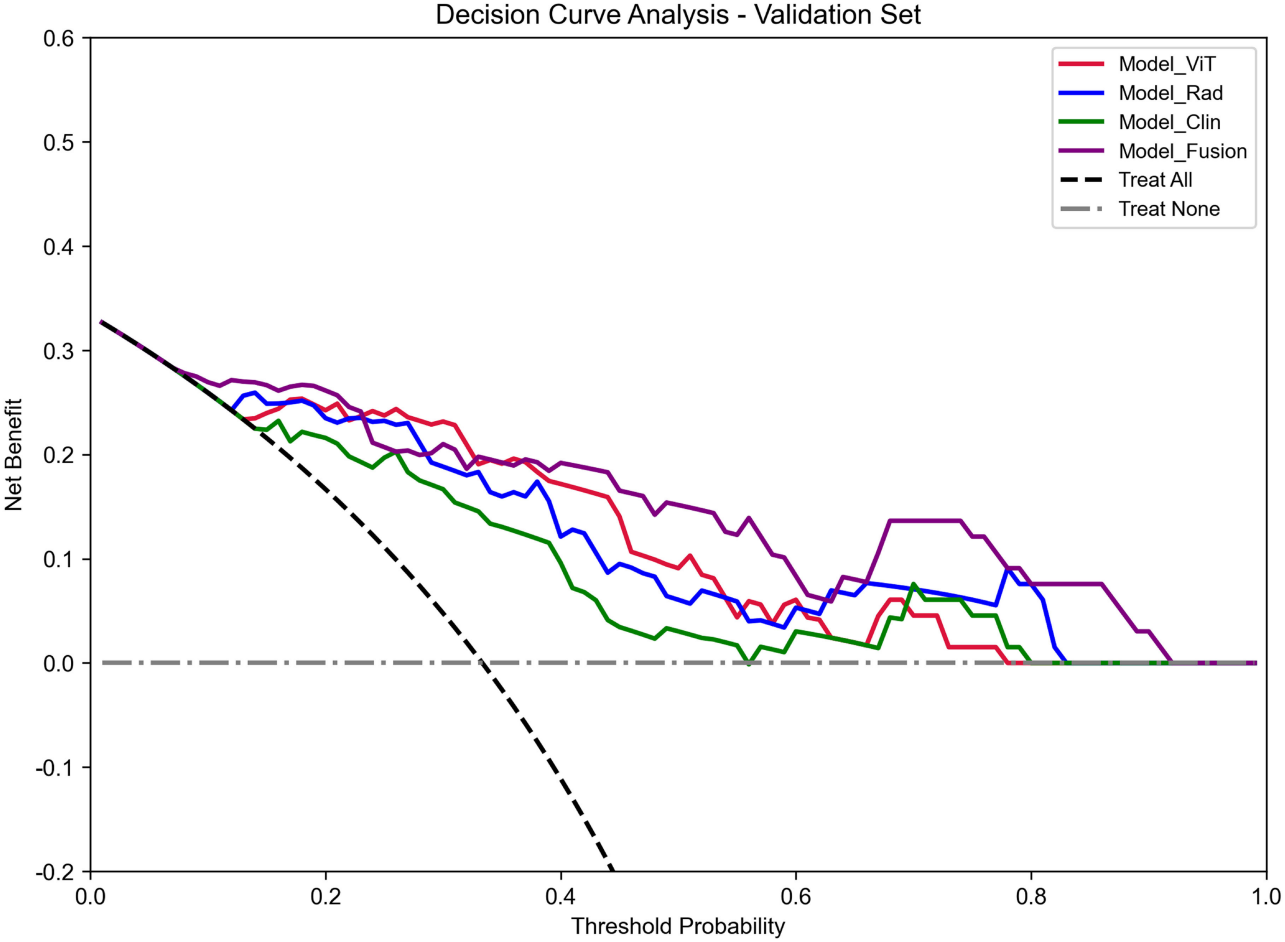
DCA curves indicate that the Model_Fusion has higher clinical utility.

**Figure 8:**
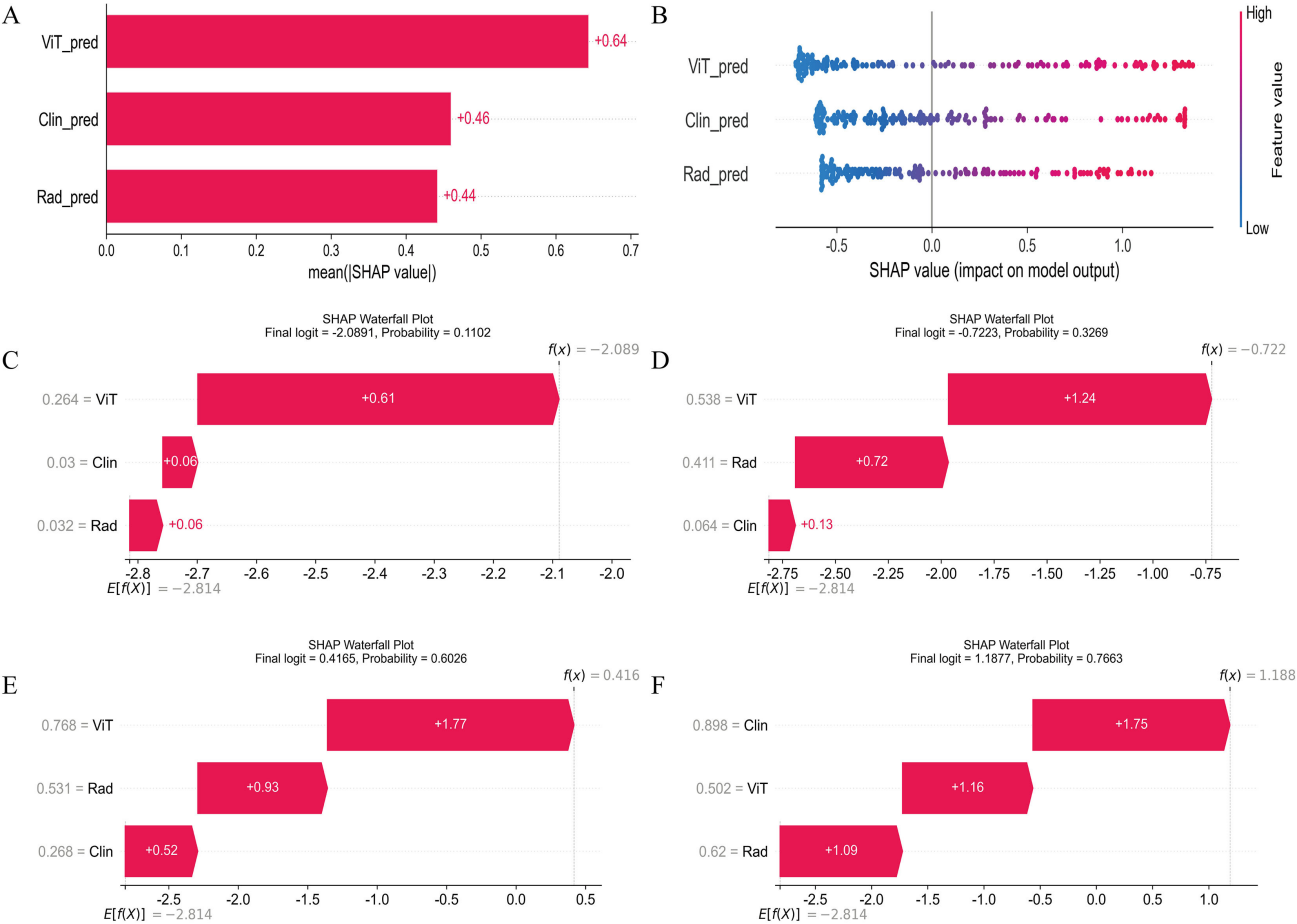
SHAP analysis of Model_Fusion. **(A, B)** Feature importance ranking and summary plots showing that ViT contributed the most to the multimodal decision-making process and played a leading role; **(C, D)** waterfall plots for two patients without BM; **(E, F)** waterfall plots for two patients with BM. ViT, Vision Transformer; BM, bone metastasis.

To evaluate the predictive performance of multimodal integration, we combined the predicted probabilities from Model_Clin, Model_Rad, and Model_ViT to construct Model_Fusion, which further improved predictive performance. The ROC curves (**Figure 4**) showed that the AUCs of Model_Fusion were 0.944 (0.902–0.977) in the training set and 0.894 (0.809–0.959) in the validation set. The AUC results for all models are detailed in **Table 4**. DeLong test results (**Figure 5**) showed that, in the training set, Model_Fusion had a significantly higher AUC than Model_Clin (*P* = 0.004), Model_Rad (*P* = 0.011), and Model_ViT (*P* = 0.023), whereas a significant difference was observed only when compared with Model_Clin (*P* = 0.008) in the validation set. Calibration curves (**Figure 6**) demonstrated that Model_Fusion had strong consistency between predicted and actual outcomes. DCA results (**Figure 7**) showed that within the relevant range of threshold probabilities, Model_Fusion provided a higher net benefit, suggesting its clinical utility. SHAP analysis (**Figure 8**) demonstrated that ViT contributed the most to the multimodal decision-making process, while waterfall plots further illustrated distinct feature contribution patterns between patients with and without BM.

**Table 4:** Performance of the models in the training and validation sets.

Model	AUC	Accuracy	Sensitivity	Specificity	PPV	NPV	F1-score
Training set							
Model_Clin	0.861 (0.791–0.924)	0.844	0.580	0.971	0.906	0.828	0.707
Model_Rad	0.885 (0.825–0.937)	0.812	0.580	0.923	0.784	0.821	0.667
Model_ViT	0.909 (0.855–0.955)	0.851	0.740	0.904	0.787	0.879	0.763
Model_Fusion	0.944 (0.902–0.977)	0.877	0.720	0.952	0.878	0.876	0.791
Validation set							
Model_Clin	0.781 (0.661–0.884)	0.697	0.318	0.886	0.583	0.722	0.412
Model_Rad	0.842 (0.736–0.928)	0.727	0.455	0.864	0.625	0.760	0.526
Model_ViT	0.872 (0.783–0.943)	0.758	0.545	0.864	0.667	0.792	0.600
Model_Fusion	0.894 (0.809–0.959)	0.818	0.636	0.909	0.778	0.833	0.700

AUC, area under the curve; NPV, negative predictive value; PPV, positive predictive value.

## 4 Discussion

BM is the most common metastatic manifestation in PCa, primarily affecting the pelvis and spine. PCa patients with BM have poorer prognoses and higher mortality rates (17). The presence of BM directly influences clinical decision-making and patient prognosis. Therefore, accurate identification of BM status is of great importance. Radiomics enables the quantitative analysis of medical imaging data using computational methods. Previous studies have demonstrated that radiomics can provide valuable information for clinical decision-making (18–20). Zhang et al. (21) reported that a radiomics model incorporating 12 features could effectively predict BM status. Furthermore, the integration of radiomics with clinical features can further improve predictive performance (22). Liu et al. (23) constructed a radiomics model based on both intratumoral and peritumoral regions from dual-parameter MR images to predict bone metastasis in newly diagnosed prostate cancer, demonstrating that the inclusion of peritumoral features enhanced predictive performance. Although radiomics can reflect tumor heterogeneity to some extent, its ability to capture such heterogeneity remains limited by subjective factors and feature selection strategies (24).

ViT is an emerging model in the field of deep learning, inspired by the Transformer architecture originally developed for natural language processing (25). ViT applies the transformer architecture to computer vision tasks by dividing input images into fixed-size patches and mapping them to fixed-dimensional vectors. Subsequently, these vectors are input into the Transformer for feature extraction and learning (26). The ViT model has several advantages. First, it exhibits strong scalability, with performance improving as model size and training data increase. Second, compared with traditional convolutional neural networks, ViT does not rely on convolution operations but directly uses the self-attention mechanism to model the image, thereby enhancing its ability to capture global information. Although ViT has made significant progress in natural image analysis, its application in medical image analysis remains at an early stage. Zhang et al. (27) proposed a ViT-based model using DCE-MRI for non-invasive classification of HER2-zero, HER2-low, and HER2-positive breast cancer, which demonstrated stable performance in an external test cohort, with AUCs of 0.80, 0.73, and 0.71, respectively. Ayana et al. (28) proposed a ViT-based approach for breast ultrasound image classification, employing a multi-stage transfer-learning strategy in which the model was pre-trained on ImageNet and cancer cell image datasets and subsequently fine-tuned for the target task, achieving promising performance. Zhang et al. (29) developed a multimodal fusion framework integrating ViT, habitat analysis, and radiomics to predict the response to androgen deprivation therapy in PCa, achieving the best performance with an AUC of 0.886; SHAP analysis showed that the ViT model contributed the most. In this study, the Model_ViT was trained on 2.5D regions, improving predictive performance by preserving rich spatial contextual information. Its AUC values in the training and validation sets were 0.909 and 0.872, respectively, outperforming Model_Rad and Model_Clin. Traditional radiomics mainly relies on artificially defined intensity, texture and shape features. Although these features offer a degree of interpretability, their ability to depict complex non-linear patterns and high-order spatial information is limited (30). In contrast, ViT can automatically learn deeper image features through end-to-end training and capture the dependencies between local and global aspects, thereby more fully exploring the heterogeneous phenotypes associated with BM in the image (31). It is worth noting that this study achieved favorable performance using only the ADC sequence. ADC reflects the degree of water-molecule diffusion restriction and is associated with tumor cell density and tissue microstructural characteristics. Tumors with a higher propensity for BM often exhibit more aggressive biological behavior, which may be partially captured by ADC-derived imaging features (32). Lower ADC values generally indicate increased cellularity and microstructural complexity, both of which have been linked to more aggressive tumor phenotypes. Therefore, ADC-derived functional information may provide a useful imaging biomarker for assessing BM status.

In this study, a multimodal fusion model integrating ViT, radiomics, and clinical features was constructed as a non-invasive framework for predicting BM status in PCa. Model_Fusion achieved good performance (AUC = 0.944 and 0.894) in the training and validation sets, respectively. Compared with single-modal models, Model_Fusion showed numerically higher performance, suggesting that integrating multiple sources of information may provide complementary information for prediction. Specifically, deep features extracted by the ViT model possess strong non-linear representation capabilities, while radiomics features capture tumor texture and shape characteristics. In addition, clinical features provide essential background information related to the biological behavior of the disease. The integration of these modalities enhanced the ability of Model_Fusion to predict BM status. It is worth noting that the DeLong test indicated that the differences between Model_Fusion and the other models in the training set were statistically significant. Although Model_Fusion achieved the highest AUC and F1-score in the validation set, its advantage over Model_ViT and Model_Rad was limited and did not reach statistical significance; a statistically significant difference was observed only when compared with Model_Clin. Therefore, the superiority of Model_Fusion over Model_ViT and Model_Rad should be interpreted with caution. Nevertheless, Model_Fusion consistently showed numerically higher values across multiple evaluation metrics, which may indicate that multimodal integration provides complementary information. The higher F1-score indicates a better balance between false positives and false negatives, while the AUC reflects overall discriminative ability across thresholds (33). Overall, Model_Fusion maintained stable performance under class imbalance, suggesting potential clinical utility, although further validation in larger external cohorts is required. The DCA results indicated that, when the threshold probability was within the relevant range, Model_Fusion could provide higher net benefits for clinical decision-making. SHAP analysis was employed to explain the Model_Fusion, and the results indicated that in the multimodal decision-making process, Model_Fusion had the highest dependence on the ViT; thus, its contribution was more significant in predicting BM status.

This study has several limitations. First, this study was a single-center retrospective study with a relatively small sample size, which may have limited the model’s ability to fully learn the data distribution and may have affected the stability of the results. Second, the model was constructed and validated using only single-center data, without independent external validation across different centers, scanners, and populations. Future studies involving larger, prospective, multicenter cohorts are warranted to further assess the robustness, generalizability, and clinical utility of the model. Third, manual ROI segmentation is inefficient and dependent on physician experience, which may limit scalability. The adoption of automated or semi-automated segmentation methods will be key for broader implementation. Fourth, the interpretability of the ViT still requires improvement. In clinical applications, medical staff and patients often require clearer decision-making bases. Therefore, future research should focus on enhancing interpretability and promoting the effective application of ViT-based models into clinical processes.

## 5 Conclusion

In conclusion, our study demonstrates that a fusion model integrating ViT, radiomics, and clinical features provides a non-invasive framework for predicting BM status in PCa, which may help guide clinical decision-making and prognostic assessment.

## Data Availability

The original contributions presented in the study are included in the article/**Supplementary Material**. Further inquiries can be directed to the corresponding authors.
