# Supplementary material for: A multimodal fusion model integrating Vision Transformer, radiomics, and clinical features for predicting bone metastasis in prostate cancer
Source: Front Oncol. 2026 Jul 6;16:1841761. doi: 10.3389/fonc.2026.1841761 (PMC13381310; doi:10.3389/fonc.2026.1841761)
Supplement: Supplementary file 3 [file DataSheet3.docx]

**A multimodal fusion model integrating vision transformer, radiomics, and clinical features for prediction of bone metastasis in prostate cancer**

**supplementary materials**

**Radiomics feature extraction**

Radiomics feature extraction including first-order statistics, shape-based features, and second-order texture features—namely the gray level run length matrix (GLRLM), gray level co-occurrence matrix (GLCM), gray level dependence matrix (GLDM), neighboring gray tone difference matrix (NGTDM), and gray level size zone matrix (GLSZM) and higher-order features derived from wavelet transformations and Laplacian of Gaussian (LoG) filtering.

**The calculation formula for the Rad-score is as follows**

Rad-score=-0.0085 × original_shape_Sphericity

－ 0.0073 × original_firstorder_Minimum

＋ 0.0291 × original_glcm_Correlation

－ 0.0156 × original_glszm_SizeZoneNonUniformityNormalized

－ 0.0274 × log-sigma-2-mm-3D_glszm_SmallAreaLowGrayLevelEmphasis

＋ 0.0686 × log-sigma-3-mm-3D_glcm_ClusterShade

＋ 0.1117 × wavelet-LLH_gldm_LargeDependenceHighGrayLevelEmphasis

＋ 0.0440 × wavelet-LHH_firstorder_Mean

－ 0.0118 × wavelet-LHH_gldm_LargeDependenceLowGrayLevelEmphasis

－ 0.0121 × wavelet-LHH_ngtdm_Strength

－ 0.0080 × wavelet-HHL_glszm_ZonePercentage

－ 0.0258 × wavelet-HHH_gldm_DependenceVariance

＋ 0.0074 × wavelet-HHH_gldm_GrayLevelVariance

**Training parameters of the ViT model**

We have added the following details. We employed the ViT-Small/16 model (vit_small_patch16_224) from the timm library as the backbone, initialized with ImageNet-pretrained weights. The input consisted of three-channel images constructed from the largest tumor slice and its adjacent slices, resized to 224 × 224 pixels. During training, the backbone was initially frozen, and only the feature integration module (neck) and classifier were optimized. The neck comprised a fully connected layer followed by a ReLU activation and Dropout (p = 0.3), and the classifier was a fully connected layer for binary classification. After 3 epochs, the last four Transformer blocks, along with their corresponding normalization layers, were gradually unfrozen for joint fine-tuning. To address class imbalance, we used a weighted cross-entropy loss with weights computed from class frequencies. Optimization was performed using AdamW, with a learning rate of 1×10⁻⁴ for the classifier and 3×10⁻⁵ for the backbone, and a weight decay of 1×10⁻⁴. A warm-up strategy was applied during the first 3 epochs, followed by cosine annealing with a minimum learning rate of 5% of the initial value. Training was conducted for up to 50 epochs with a batch size of 4, and early stopping (patience = 5) was employed. Label smoothing (0.05) and gradient clipping (max norm = 1.0) were applied to improve training stability. Data augmentation included random horizontal flip (p = 0.5), vertical flip (p = 0.2), rotation (±10°), affine transformation (p = 0.3; translation = 0.03, scale = 0.95–1.05), and Gaussian blur (p = 0.2). No random augmentation was applied during validation. During model development, five-fold cross-validation was performed on the training set. In each fold, the model was trained on the training subset and evaluated on the validation subset, with model selection based on AUC of the validation subset. Early stopping is applied if the validation performance does not improve for five consecutive epochs. The optimal classification threshold was determined by maximizing the Youden index on the validation subset. The thresholds obtained from all folds were then averaged to derive a single, robust threshold. After model development, a final model was retrained on the full training set and evaluated on an internal validation set that was not used at any stage of model development. For reproducibility, a fixed random seed (seed = 42) was used.

Table 1 Performance evaluation of different models in five-fold cross-validation

| Model | AUC | ACC | SEN | SPC | PPV | NPV | F1-score |
| --- | --- | --- | --- | --- | --- | --- | --- |
| Fold 1 | | | | | | | |
| Model_Rad | 0.902 | 0.839 | 0.625 | 0.913 | 0.714 | 0.875 | 0.667 |
| Model_ViT | 0.918 | 0.871 | 0.750 | 0.913 | 0.750 | 0.913 | 0.750 |
| Model_Fusion | 0.955 | 0.903 | 0.750 | 0.957 | 0.857 | 0.917 | 0.800 |
| Fold 2 | | | | | | | |
| Model_Rad | 0.856 | 0.774 | 0.583 | 0.895 | 0.778 | 0.773 | 0.667 |
| Model_ViT | 0.875 | 0.806 | 0.667 | 0.895 | 0.800 | 0.810 | 0.727 |
| Model_Fusion | 0.921 | 0.871 | 0.750 | 0.947 | 0.900 | 0.857 | 0.818 |
| Fold 3 | | | | | | | |
| Model_Rad | 0.925 | 0.871 | 0.700 | 0.952 | 0.875 | 0.870 | 0.778 |
| Model_ViT | 0.940 | 0.903 | 0.800 | 0.952 | 0.889 | 0.909 | 0.842 |
| Model_Fusion | 0.963 | 0.903 | 0.800 | 0.952 | 0.889 | 0.909 | 0.842 |
| Fold 4 | | | | | | | |
| Model_Rad | 0.813 | 0.733 | 0.444 | 0.857 | 0.571 | 0.783 | 0.500 |
| Model_ViT | 0.842 | 0.800 | 0.667 | 0.857 | 0.667 | 0.857 | 0.667 |
| Model_Fusion | 0.899 | 0.833 | 0.667 | 0.905 | 0.750 | 0.864 | 0.706 |
| Fold 5 | | | | | | | |
| Model_Rad | 0.875 | 0.774 | 0.545 | 0.900 | 0.750 | 0.783 | 0.632 |
| Model_ViT | 0.892 | 0.839 | 0.727 | 0.900 | 0.800 | 0.857 | 0.762 |
| Model_Fusion | 0.932 | 0.871 | 0.727 | 0.950 | 0.889 | 0.864 | 0.800 |
| Average results | | | | | | | |
| Model_Rad | 0.874 ±0.040 | 0.798 ± 0.053 | 0.579 ± 0.095 | 0.903 ± 0.035 | 0.738 ± 0.108 | 0.817 ± 0.044 | 0.649 ± 0.105 |
| Model_ViT | 0.893 ± 0.035 | 0.844 ± 0.042 | 0.722 ± .0500 | 0.903 ± 0.035 | 0.781 ± 0.086 | 0.869 ± 0.036 | 0.750 ± 0.065 |
| Model_Fusion | 0.934 ± 0.026 | 0.876 ± 0.027 | 0.739 ±0.035 | 0.942 ± 0.022 | 0.857 ± 0.060 | 0.882 ± 0.028 | 0.793 ± 0.052 |
