# Supplementary material for: A multimodal fusion model integrating Vision Transformer, radiomics, and clinical features for predicting bone metastasis in prostate cancer
Source: Front Oncol. 2026 Jul 6;16:1841761. doi: 10.3389/fonc.2026.1841761 (PMC13381310; doi:10.3389/fonc.2026.1841761)
Supplement: Supplementary file 4 [file Table1.doc]

import os

import copy

import math

import random

import warnings

import numpy as np

import pandas as pd

from sklearn.metrics import (

roc_auc_score,

accuracy_score,

precision_score,

f1_score,

confusion_matrix,

roc_curve,

)

import torch

import torch.nn as nn

from torch.utils.data import DataLoader, WeightedRandomSampler

from torch.cuda.amp import autocast, GradScaler

from torchvision import transforms

from torchvision.transforms import functional as TF

from torchvision.transforms import InterpolationMode

from src.config import Config

from src.dataset import ProstateMRIDataset

from src.model import ViTModel

warnings.filterwarnings("ignore")

# =========================

# 0. torchvision 旧版本兼容封装

# =========================

def safe_resize(image_size):

"""

torchvision 新版支持 antialias 参数，旧版不支持。

"""

try:

return transforms.Resize(

(image_size, image_size),

interpolation=InterpolationMode.BILINEAR,

antialias=True

)

except TypeError:

return transforms.Resize(

(image_size, image_size),

interpolation=InterpolationMode.BILINEAR

)

def safe_random_rotation(degrees, interpolation=InterpolationMode.BILINEAR):

"""

RandomRotation 新版支持 interpolation，旧版可能不支持该关键字参数。

"""

try:

return transforms.RandomRotation(degrees=degrees, interpolation=interpolation)

except TypeError:

# 旧版：不传 interpolation（或使用 resample，但不同版本签名差异更大）

return transforms.RandomRotation(degrees=degrees)

def safe_random_affine(

degrees=0,

translate=(0.03, 0.03),

scale=(0.95, 1.05),

interpolation=InterpolationMode.BILINEAR

):

"""

RandomAffine 新版支持 interpolation，旧版可能不支持。

"""

try:

return transforms.RandomAffine(

degrees=degrees,

translate=translate,

scale=scale,

interpolation=interpolation

)

except TypeError:

return transforms.RandomAffine(

degrees=degrees,

translate=translate,

scale=scale

)

def safe_gaussian_blur(kernel_size=3, sigma=(0.1, 1.0)):

"""

GaussianBlur 在非常旧的 torchvision 里可能没有。

"""

if hasattr(transforms, "GaussianBlur"):

return transforms.GaussianBlur(kernel_size=kernel_size, sigma=sigma)

else:

return transforms.Lambda(lambda x: x)

# =========================

# 1. 强复现：固定随机种子

# =========================

def set_seed(seed=42):

os.environ["PYTHONHASHSEED"] = str(seed)

os.environ["CUBLAS_WORKSPACE_CONFIG"] = ":4096:8"

random.seed(seed)

np.random.seed(seed)

torch.manual_seed(seed)

torch.cuda.manual_seed(seed)

torch.cuda.manual_seed_all(seed)

torch.backends.cudnn.deterministic = True

torch.backends.cudnn.benchmark = False

try:

torch.use_deterministic_algorithms(True)

except Exception as e:

print(f"[警告] 无法完全启用 deterministic algorithms: {e}")

def seed_worker(worker_id):

worker_seed = Config.SEED + worker_id

np.random.seed(worker_seed)

random.seed(worker_seed)

torch.manual_seed(worker_seed)

def ensure_dir(path):

os.makedirs(path, exist_ok=True)

# =========================

# 2. 工具函数

# =========================

def safe_read_csv(csv_path):

encodings = ["utf-8", "utf-8-sig", "gbk", "gb2312", "latin1"]

last_err = None

for enc in encodings:

try:

return pd.read_csv(csv_path, encoding=enc)

except Exception as e:

last_err = e

raise last_err

def validate_fixed_split_csv(csv_path):

df = safe_read_csv(csv_path)

df.columns = df.columns.astype(str).str.strip().str.lower()

required_columns = ["label", "name", "split"]

missing = [c for c in required_columns if c not in df.columns]

if missing:

raise ValueError(f"CSV 缺少必要列: {missing}")

df["split"] = df["split"].astype(str).str.strip().str.lower()

valid_values = {"train", "val"}

bad_values = set(df["split"].unique()) - valid_values

if len(bad_values) > 0:

raise ValueError(f"split 列只能包含 train / val，发现非法取值: {bad_values}")

if df["name"].duplicated().any():

dup_names = df.loc[df["name"].duplicated(), "name"].tolist()

raise ValueError(f"CSV 中存在重复 name，请保证唯一。重复示例: {dup_names[:5]}")

return df

def prepare_fixed_split_csvs(output_dir):

"""

固定分组：

从一个总 CSV 中读取 label / name / split

split 只能是 train / val

"""

ensure_dir(output_dir)

csv_path = Config.CSV_PATH

df = validate_fixed_split_csv(csv_path)

train_df = df[df["split"] == "train"].copy().reset_index(drop=True)

val_df = df[df["split"] == "val"].copy().reset_index(drop=True)

if len(train_df) == 0:

raise ValueError("train 集为空，请检查 split 列。")

if len(val_df) == 0:

raise ValueError("val 集为空，请检查 split 列。")

train_csv = os.path.join(output_dir, "train_split.csv")

val_csv = os.path.join(output_dir, "val_split.csv")

train_df.to_csv(train_csv, index=False, encoding="utf-8-sig")

val_df.to_csv(val_csv, index=False, encoding="utf-8-sig")

print("使用固定分组，不进行随机划分。")

print(f"训练集数量: {len(train_df)}")

print(f"验证集数量: {len(val_df)}")

print("训练集标签分布:")

print(train_df["label"].value_counts().sort_index())

print("验证集标签分布:")

print(val_df["label"].value_counts().sort_index())

overlap = set(train_df["name"]).intersection(set(val_df["name"]))

print(f"train/val 名称重叠数: {len(overlap)}")

if len(overlap) > 0:

raise ValueError("固定分组错误：train 和 val 中存在重复 name。")

return train_csv, val_csv

def freeze_backbone_parameters(model):

"""

只冻结 backbone，保留 neck + classifier 可训练

适配当前自定义 ViTModel:

model.backbone

model.neck

model.classifier

"""

if not hasattr(model, "backbone"):

raise ValueError("模型中未找到 backbone，请检查 src/model.py")

for param in model.backbone.parameters():

param.requires_grad = False

if hasattr(model, "neck"):

for param in model.neck.parameters():

param.requires_grad = True

if hasattr(model, "classifier"):

for param in model.classifier.parameters():

param.requires_grad = True

total_params = sum(p.numel() for p in model.parameters())

trainable_params = sum(p.numel() for p in model.parameters() if p.requires_grad)

print(f"[Freeze Backbone] 总参数量: {total_params:,}")

print(f"[Freeze Backbone] 可训练参数量: {trainable_params:,}")

print("[Freeze Backbone] 当前可训练参数：")

found = False

for name, param in model.named_parameters():

if param.requires_grad:

print(f" - {name}: {param.numel():,}")

found = True

if not found:

print(" [警告] 没有找到可训练参数，请检查模型结构。")

def unfreeze_last_n_blocks(model, n_blocks=4):

"""

分阶段解冻：

只解冻 ViT backbone 的最后 n 个 block + norm

neck / classifier 始终保持可训练

"""

if not hasattr(model, "backbone"):

raise ValueError("模型中未找到 backbone")

for param in model.backbone.parameters():

param.requires_grad = False

if hasattr(model.backbone, "blocks"):

blocks = model.backbone.blocks

n_blocks = min(n_blocks, len(blocks))

for block in blocks[-n_blocks:]:

for param in block.parameters():

param.requires_grad = True

else:

print("[警告] 当前 backbone 未找到 blocks 属性，无法按 block 解冻。")

if hasattr(model.backbone, "norm"):

for param in model.backbone.norm.parameters():

param.requires_grad = True

if hasattr(model, "neck"):

for param in model.neck.parameters():

param.requires_grad = True

if hasattr(model, "classifier"):

for param in model.classifier.parameters():

param.requires_grad = True

total_params = sum(p.numel() for p in model.parameters())

trainable_params = sum(p.numel() for p in model.parameters() if p.requires_grad)

print(f"[Unfreeze] 总参数量: {total_params:,}")

print(f"[Unfreeze] 可训练参数量: {trainable_params:,}")

print(f"[Unfreeze] 已解冻 backbone 最后 {n_blocks} 个 block")

def get_optimizer_params(model):

return [p for p in model.parameters() if p.requires_grad]

def build_optimizer(model, head_lr, backbone_lr, weight_decay):

"""

neck + classifier 用 head_lr

已解冻的 backbone 参数用 backbone_lr

"""

backbone_params = []

head_params = []

for name, param in model.named_parameters():

if not param.requires_grad:

continue

if "backbone" in name:

backbone_params.append(param)

else:

head_params.append(param)

param_groups = []

if len(backbone_params) > 0:

param_groups.append({"params": backbone_params, "lr": backbone_lr})

if len(head_params) > 0:

param_groups.append({"params": head_params, "lr": head_lr})

optimizer = torch.optim.AdamW(

param_groups,

weight_decay=weight_decay,

)

return optimizer

def build_warmup_cosine_scheduler(optimizer, warmup_epochs, total_epochs, min_lr_ratio=0.05):

"""

epoch 级别的 warmup + cosine

"""

def lr_lambda(current_epoch):

if current_epoch < warmup_epochs:

return float(current_epoch + 1) / float(max(1, warmup_epochs))

progress = float(current_epoch - warmup_epochs) / float(max(1, total_epochs - warmup_epochs))

progress = min(max(progress, 0.0), 1.0)

cosine = 0.5 * (1.0 + math.cos(math.pi * progress))

return min_lr_ratio + (1.0 - min_lr_ratio) * cosine

scheduler = torch.optim.lr_scheduler.LambdaLR(optimizer, lr_lambda=lr_lambda)

return scheduler

def find_best_threshold(y_true, y_prob):

"""

基于 Youden Index 搜索最佳阈值

"""

y_true = np.asarray(y_true).astype(int)

y_prob = np.asarray(y_prob).astype(float)

if len(np.unique(y_true)) < 2:

return 0.5

fpr, tpr, thresholds = roc_curve(y_true, y_prob)

youden = tpr - fpr

best_idx = np.argmax(youden)

best_thr = thresholds[best_idx]

if not np.isfinite(best_thr):

best_thr = 0.5

best_thr = max(0.0, min(1.0, float(best_thr)))

return best_thr

# =========================

# 3. 图像预处理 / 增强

# =========================

class EnsureTensorFloat:

def __call__(self, x):

"""

统一把输入转成 float32 的 torch.Tensor，并保证格式为 [C, H, W]

支持输入:

- numpy.ndarray: [H,W], [H,W,C], [C,H,W]

- torch.Tensor: [H,W], [H,W,C], [C,H,W]

- PIL.Image

"""

if torch.is_tensor(x):

x = x.float()

# 若是 [H, W]，补成 [1, H, W]

if x.dim() == 2:

x = x.unsqueeze(0)

# 若是 [H, W, C]，转成 [C, H, W]

elif x.dim() == 3:

# 经验判断：如果最后一维像通道数(1/3/4)，则视为 HWC

if x.shape[-1] in [1, 3, 4] and x.shape[0] not in [1, 3, 4]:

x = x.permute(2, 0, 1).contiguous()

else:

raise ValueError(f"不支持的张量维度: {x.shape}")

if x.max() > 1.0:

x = x / 255.0

return x

# numpy / PIL 走这里

if isinstance(x, np.ndarray):

x = torch.from_numpy(x).float()

if x.dim() == 2:

x = x.unsqueeze(0)

elif x.dim() == 3:

# [H,W,C] -> [C,H,W]

if x.shape[-1] in [1, 3, 4]:

x = x.permute(2, 0, 1).contiguous()

# 否则默认已经是 [C,H,W]

else:

raise ValueError(f"不支持的 numpy 维度: {x.shape}")

if x.max() > 1.0:

x = x / 255.0

return x

# PIL.Image 等

x = TF.to_tensor(x).float()

return x

class RepeatChannelsIfNeeded:

def __init__(self, in_chans=3):

self.in_chans = in_chans

def __call__(self, x):

if not torch.is_tensor(x):

x = TF.to_tensor(x).float()

# 再保险：保证是 [C,H,W]

if x.dim() == 2:

x = x.unsqueeze(0)

elif x.dim() == 3:

if x.shape[-1] in [1, 3, 4] and x.shape[0] not in [1, 3, 4]:

x = x.permute(2, 0, 1).contiguous()

else:

raise ValueError(f"期望输入图像维度为 [C,H,W]，实际得到: {x.shape}")

c = x.shape[0]

if c == self.in_chans:

return x

if c == 1 and self.in_chans == 3:

return x.repeat(3, 1, 1)

if c == 1 and self.in_chans == 4:

return x.repeat(4, 1, 1)

if c == 3 and self.in_chans == 1:

return x[:1, :, :]

if c == 4 and self.in_chans == 3:

return x[:3, :, :]

if c == 4 and self.in_chans == 1:

return x[:1, :, :]

raise ValueError(f"通道数不匹配：当前图像通道数={c}，Config.IN_CHANNELS={self.in_chans}")

def build_transforms():

image_size = getattr(Config, "IMAGE_SIZE", 224)

in_chans = getattr(Config, "IN_CHANNELS", 3)

mean = getattr(Config, "NORMALIZE_MEAN", [0.5] * in_chans)

std = getattr(Config, "NORMALIZE_STD", [0.5] * in_chans)

use_augment = getattr(Config, "USE_AUGMENT", True)

rotate_degrees = getattr(Config, "ROTATE_DEGREES", 10)

hflip_prob = getattr(Config, "HFLIP_PROB", 0.5)

vflip_prob = getattr(Config, "VFLIP_PROB", 0.2)

affine_prob = getattr(Config, "AFFINE_PROB", 0.3)

blur_prob = getattr(Config, "BLUR_PROB", 0.2)

if use_augment:

train_transform = transforms.Compose([

EnsureTensorFloat(),

RepeatChannelsIfNeeded(in_chans=in_chans),

safe_resize(image_size),

transforms.RandomHorizontalFlip(p=hflip_prob),

transforms.RandomVerticalFlip(p=vflip_prob),

safe_random_rotation(degrees=rotate_degrees, interpolation=InterpolationMode.BILINEAR),

transforms.RandomApply([

safe_random_affine(

degrees=0,

translate=(0.03, 0.03),

scale=(0.95, 1.05),

interpolation=InterpolationMode.BILINEAR

)

], p=affine_prob),

transforms.RandomApply([

safe_gaussian_blur(kernel_size=3, sigma=(0.1, 1.0))

], p=blur_prob),

transforms.Normalize(mean=mean, std=std),

])

else:

train_transform = transforms.Compose([

EnsureTensorFloat(),

RepeatChannelsIfNeeded(in_chans=in_chans),

safe_resize(image_size),

transforms.Normalize(mean=mean, std=std),

])

val_transform = transforms.Compose([

EnsureTensorFloat(),

RepeatChannelsIfNeeded(in_chans=in_chans),

safe_resize(image_size),

transforms.Normalize(mean=mean, std=std),

])

return train_transform, val_transform

# =========================

# 4. WeightedRandomSampler

# =========================

def build_weighted_sampler(csv_path):

df = safe_read_csv(csv_path)

df.columns = df.columns.astype(str).str.strip().str.lower()

labels = df["label"].astype(int).values

class_counts = np.bincount(labels)

class_weights = 1.0 / np.maximum(class_counts, 1)

sample_weights = class_weights[labels]

sample_weights = torch.as_tensor(sample_weights, dtype=torch.double)

sampler = WeightedRandomSampler(

weights=sample_weights,

num_samples=len(sample_weights),

replacement=True,

)

return sampler

# =========================

# 5. DataLoader

# =========================

def build_dataloader(train_csv, val_csv):

train_transform, val_transform = build_transforms()

train_dataset = ProstateMRIDataset(

csv_path=train_csv,

transform=train_transform,

)

# 训练集评估单独用无增强版本，避免 train AUC 被随机增强扰动

train_eval_dataset = ProstateMRIDataset(

csv_path=train_csv,

transform=val_transform,

)

val_dataset = ProstateMRIDataset(

csv_path=val_csv,

transform=val_transform,

)

g = torch.Generator()

g.manual_seed(Config.SEED)

use_weighted_sampler = getattr(Config, "USE_WEIGHTED_SAMPLER", False)

persistent_workers = True if getattr(Config, "NUM_WORKERS", 0) > 0 else False

if use_weighted_sampler:

train_sampler = build_weighted_sampler(train_csv)

train_loader = DataLoader(

train_dataset,

batch_size=Config.BATCH_SIZE,

sampler=train_sampler,

shuffle=False,

num_workers=Config.NUM_WORKERS,

pin_memory=True,

worker_init_fn=seed_worker,

generator=g,

drop_last=False,

persistent_workers=persistent_workers,

)

print("训练集使用 WeightedRandomSampler")

else:

train_loader = DataLoader(

train_dataset,

batch_size=Config.BATCH_SIZE,

shuffle=True,

num_workers=Config.NUM_WORKERS,

pin_memory=True,

worker_init_fn=seed_worker,

generator=g,

drop_last=False,

persistent_workers=persistent_workers,

)

print("训练集使用普通 shuffle")

train_eval_loader = DataLoader(

train_eval_dataset,

batch_size=Config.BATCH_SIZE,

shuffle=False,

num_workers=Config.NUM_WORKERS,

pin_memory=True,

worker_init_fn=seed_worker,

generator=g,

drop_last=False,

persistent_workers=persistent_workers,

)

val_loader = DataLoader(

val_dataset,

batch_size=Config.BATCH_SIZE,

shuffle=False,

num_workers=Config.NUM_WORKERS,

pin_memory=True,

worker_init_fn=seed_worker,

generator=g,

drop_last=False,

persistent_workers=persistent_workers,

)

return train_loader, train_eval_loader, val_loader

# =========================

# 6. Loss

# =========================

class LabelSmoothingCrossEntropy(nn.Module):

def __init__(self, weight=None, smoothing=0.0):

super().__init__()

self.weight = weight

self.smoothing = smoothing

def forward(self, logits, target):

num_classes = logits.size(1)

if num_classes <= 1:

raise ValueError("num_classes 必须大于 1")

log_probs = torch.log_softmax(logits, dim=1)

with torch.no_grad():

true_dist = torch.zeros_like(log_probs)

true_dist.fill_(self.smoothing / (num_classes - 1))

true_dist.scatter_(1, target.unsqueeze(1), 1.0 - self.smoothing)

if self.weight is not None:

class_weight = self.weight[target].unsqueeze(1)

loss = (-true_dist * log_probs).sum(dim=1, keepdim=True) * class_weight

return loss.mean()

loss = (-true_dist * log_probs).sum(dim=1)

return loss.mean()

def calculate_class_weights(csv_path):

df = safe_read_csv(csv_path)

df.columns = df.columns.astype(str).str.strip().str.lower()

counts = df["label"].value_counts().sort_index()

n0 = counts.get(0, 0)

n1 = counts.get(1, 0)

total = n0 + n1

if n0 == 0 or n1 == 0:

return torch.tensor([1.0, 1.0], dtype=torch.float32)

w0 = total / (2.0 * n0)

w1 = total / (2.0 * n1)

return torch.tensor([w0, w1], dtype=torch.float32)

# =========================

# 7. 概率提取

# =========================

def extract_positive_prob(outputs, logits):

"""

兼容以下情况：

1) outputs["prob"] 是 [B] 的正类概率

2) outputs["prob"] 是 [B, 2] 的各类概率

3) 没有 prob，则从 logits 计算

"""

prob = outputs.get("prob", None)

if prob is None:

if logits.size(1) == 2:

prob = torch.softmax(logits, dim=1)[:, 1]

elif logits.size(1) == 1:

prob = torch.sigmoid(logits[:, 0])

else:

raise ValueError("无法从 logits 推断二分类正类概率，请检查模型输出。")

return prob

if prob.dim() == 1:

return prob

if prob.dim() == 2 and prob.size(1) == 2:

return prob[:, 1]

if prob.dim() == 2 and prob.size(1) == 1:

return prob[:, 0]

raise ValueError(f"无法识别 outputs['prob'] 的形状: {prob.shape}")

# =========================

# 8. 指标

# =========================

def compute_metrics(y_true, y_prob, threshold=0.5):

y_true = np.asarray(y_true).astype(int)

y_prob = np.asarray(y_prob).astype(float)

y_pred = (y_prob >= threshold).astype(int)

auc = roc_auc_score(y_true, y_prob) if len(np.unique(y_true)) > 1 else 0.0

acc = accuracy_score(y_true, y_pred)

precision = precision_score(y_true, y_pred, zero_division=0)

f1 = f1_score(y_true, y_pred, zero_division=0)

tn, fp, fn, tp = confusion_matrix(y_true, y_pred, labels=[0, 1]).ravel()

sensitivity = tp / (tp + fn) if (tp + fn) > 0 else 0.0

specificity = tn / (tn + fp) if (tn + fp) > 0 else 0.0

return {

"auc": auc,

"acc": acc,

"precision": precision,

"f1": f1,

"sensitivity": sensitivity,

"specificity": specificity,

"tn": tn,

"fp": fp,

"fn": fn,

"tp": tp,

"threshold": threshold,

}

def save_metrics_csv(metrics, save_dir, filename):

ensure_dir(save_dir)

metrics_df = pd.DataFrame({

"Metric": [

"AUC",

"Accuracy",

"Precision",

"F1 Score",

"Sensitivity",

"Specificity",

"Threshold",

"TP",

"TN",

"FP",

"FN",

],

"Value": [

metrics["auc"],

metrics["acc"],

metrics["precision"],

metrics["f1"],

metrics["sensitivity"],

metrics["specificity"],

metrics.get("threshold", 0.5),

metrics["tp"],

metrics["tn"],

metrics["fp"],

metrics["fn"],

]

})

save_path = os.path.join(save_dir, filename)

metrics_df.to_csv(save_path, index=False, encoding="utf-8-sig")

print(f"指标结果已保存: {save_path}")

# =========================

# 9. 训练 / 评估

# =========================

def train_one_epoch(model, loader, criterion, optimizer, device, scaler, grad_clip=None):

model.train()

total_loss = 0.0

all_labels = []

all_probs = []

for batch in loader:

images = batch["image"].to(device, non_blocking=True)

labels = batch["label"].to(device, non_blocking=True).long()

optimizer.zero_grad(set_to_none=True)

with autocast(enabled=(device.type == "cuda")):

outputs = model(images)

logits = outputs["logits"]

probs = extract_positive_prob(outputs, logits)

loss = criterion(logits, labels)

scaler.scale(loss).backward()

if grad_clip is not None and grad_clip > 0:

scaler.unscale_(optimizer)

trainable_params = [

p for p in model.parameters()

if p.requires_grad and p.grad is not None

]

if len(trainable_params) > 0:

torch.nn.utils.clip_grad_norm_(trainable_params, max_norm=grad_clip)

scaler.step(optimizer)

scaler.update()

total_loss += loss.item() * images.size(0)

all_labels.extend(labels.detach().cpu().numpy())

all_probs.extend(probs.detach().cpu().numpy())

epoch_loss = total_loss / len(loader.dataset)

metrics = compute_metrics(np.array(all_labels), np.array(all_probs), threshold=Config.DEFAULT_THRESHOLD)

return epoch_loss, metrics

@torch.no_grad()

def evaluate_and_collect(model, loader, criterion, device):

model.eval()

total_loss = 0.0

all_labels = []

all_probs = []

all_names = []

all_features = []

for batch in loader:

images = batch["image"].to(device, non_blocking=True)

labels = batch["label"].to(device, non_blocking=True).long()

names = batch["name"]

with autocast(enabled=(device.type == "cuda")):

outputs = model(images)

logits = outputs["logits"]

probs = extract_positive_prob(outputs, logits)

features = outputs["feature"]

loss = criterion(logits, labels)

total_loss += loss.item() * images.size(0)

all_labels.extend(labels.detach().cpu().numpy())

all_probs.extend(probs.detach().cpu().numpy())

all_names.extend(list(names))

all_features.append(features.detach().cpu().numpy())

epoch_loss = total_loss / len(loader.dataset)

all_labels = np.array(all_labels)

all_probs = np.array(all_probs)

all_features = np.concatenate(all_features, axis=0)

metrics_default = compute_metrics(

all_labels,

all_probs,

threshold=Config.DEFAULT_THRESHOLD

)

if getattr(Config, "FIND_BEST_THRESHOLD", True):

best_threshold = find_best_threshold(all_labels, all_probs)

else:

best_threshold = Config.DEFAULT_THRESHOLD

metrics_best = compute_metrics(

all_labels,

all_probs,

threshold=best_threshold

)

return (

epoch_loss,

metrics_default,

metrics_best,

best_threshold,

all_names,

all_labels,

all_probs,

all_features,

)

# =========================

# 10. 保存结果

# =========================

def save_results(names, labels, probs, features, save_dir, prefix="best_val"):

ensure_dir(save_dir)

result_df = pd.DataFrame({

"name": names,

"label": labels,

"dl_score": probs,

})

feature_df = pd.DataFrame(features)

feature_df.insert(0, "name", names)

feature_df.insert(1, "label", labels)

result_df = result_df.sort_values("name").reset_index(drop=True)

feature_df = feature_df.sort_values("name").reset_index(drop=True)

result_csv_path = os.path.join(save_dir, f"{prefix}_predictions.csv")

feature_csv_path = os.path.join(save_dir, f"{prefix}_features.csv")

result_df.to_csv(result_csv_path, index=False, encoding="utf-8-sig")

feature_df.to_csv(feature_csv_path, index=False, encoding="utf-8-sig")

print(f"{prefix} 预测结果已保存: {result_csv_path}")

print(f"{prefix} 特征已保存: {feature_csv_path}")

# =========================

# 11. 主程序

# =========================

def main():

set_seed(Config.SEED)

output_dir = Config.OUTPUT_DIR

ensure_dir(output_dir)

print(f"SEED = {Config.SEED}")

print("已启用尽量强复现模式。")

device = torch.device("cuda" if torch.cuda.is_available() else "cpu")

print("使用设备:", device)

label_smoothing = getattr(Config, "LABEL_SMOOTHING", 0.05)

grad_clip = getattr(Config, "GRAD_CLIP", 1.0)

freeze_backbone = getattr(Config, "FREEZE_BACKBONE", True)

min_delta = getattr(Config, "MIN_DELTA", 1e-4)

use_weighted_sampler = getattr(Config, "USE_WEIGHTED_SAMPLER", False)

freeze_epochs = getattr(Config, "FREEZE_EPOCHS", 3)

unfreeze_last_n_blocks_num = getattr(Config, "UNFREEZE_LAST_N_BLOCKS", 4)

warmup_epochs = getattr(Config, "WARMUP_EPOCHS", 3)

min_lr_ratio = getattr(Config, "MIN_LR_RATIO", 0.05)

# 1. 固定 train / val 分组

train_csv, val_csv = prepare_fixed_split_csvs(output_dir)

# 2. dataloader

train_loader, train_eval_loader, val_loader = build_dataloader(train_csv, val_csv)

# 3. model

model = ViTModel(

model_name=Config.MODEL_NAME,

pretrained=Config.PRETRAINED,

num_classes=Config.NUM_CLASSES,

dropout=Config.DROPOUT,

in_chans=Config.IN_CHANNELS,

).to(device)

# 4. 冻结 backbone

if freeze_backbone:

freeze_backbone_parameters(model)

else:

print("不冻结 backbone，全量微调。")

# 5. loss

class_weights = calculate_class_weights(train_csv).to(device)

print("原始类别权重:", class_weights.detach().cpu().numpy())

print("Label smoothing:", label_smoothing)

if use_weighted_sampler:

loss_weights = None

print("当前使用 WeightedRandomSampler，因此 loss 不再额外使用 class_weights。")

else:

loss_weights = class_weights

print("当前未使用 WeightedRandomSampler，因此 loss 使用 class_weights。")

criterion = LabelSmoothingCrossEntropy(

weight=loss_weights,

smoothing=label_smoothing

)

# 6. optimizer

trainable_params = get_optimizer_params(model)

if len(trainable_params) == 0:

raise ValueError("没有可训练参数，请检查分类头是否被正确解冻。")

optimizer = build_optimizer(

model=model,

head_lr=Config.HEAD_LR,

backbone_lr=Config.BACKBONE_LR,

weight_decay=Config.WEIGHT_DECAY,

)

# 7. scheduler

scheduler = build_warmup_cosine_scheduler(

optimizer=optimizer,

warmup_epochs=warmup_epochs,

total_epochs=Config.EPOCHS,

min_lr_ratio=min_lr_ratio,

)

# 8. AMP

scaler = GradScaler(enabled=(device.type == "cuda"))

best_auc = -1.0

best_epoch = -1

best_threshold_global = Config.DEFAULT_THRESHOLD

best_model_wts = copy.deepcopy(model.state_dict())

early_stop_counter = 0

history = []

for epoch in range(Config.EPOCHS):

print(f"\n========== Epoch [{epoch + 1}/{Config.EPOCHS}] ==========")

# 分阶段解冻

if freeze_backbone and epoch == freeze_epochs:

print(f"\n[阶段切换] Epoch {epoch + 1}: 开始解冻 backbone 最后 {unfreeze_last_n_blocks_num} 个 block")

unfreeze_last_n_blocks(model, n_blocks=unfreeze_last_n_blocks_num)

optimizer = build_optimizer(

model=model,

head_lr=Config.HEAD_LR,

backbone_lr=Config.BACKBONE_LR,

weight_decay=Config.WEIGHT_DECAY,

)

scheduler = build_warmup_cosine_scheduler(

optimizer=optimizer,

warmup_epochs=warmup_epochs,

total_epochs=max(1, Config.EPOCHS - epoch),

min_lr_ratio=min_lr_ratio,

)

scaler = GradScaler(enabled=(device.type == "cuda"))

train_loss, train_metrics = train_one_epoch(

model=model,

loader=train_loader,

criterion=criterion,

optimizer=optimizer,

device=device,

scaler=scaler,

grad_clip=grad_clip,

)

(

val_loss,

val_metrics_05,

val_metrics_best,

best_threshold,

val_names,

val_labels,

val_probs,

val_features,

) = evaluate_and_collect(model, val_loader, criterion, device)

# epoch 级调度，放在每轮结束后

scheduler.step()

current_lr = optimizer.param_groups[0]["lr"]

print(

f"Train Loss: {train_loss:.4f} | "

f"AUC: {train_metrics['auc']:.4f} | "

f"ACC: {train_metrics['acc']:.4f} | "

f"SEN: {train_metrics['sensitivity']:.4f} | "

f"SPE: {train_metrics['specificity']:.4f}"

)

print(

f"Val Loss: {val_loss:.4f} | "

f"AUC: {val_metrics_best['auc']:.4f} | "

f"ACC: {val_metrics_best['acc']:.4f} | "

f"SEN: {val_metrics_best['sensitivity']:.4f} | "

f"SPE: {val_metrics_best['specificity']:.4f} | "

f"PRE: {val_metrics_best['precision']:.4f} | "

f"F1: {val_metrics_best['f1']:.4f} | "

f"Thr: {best_threshold:.4f} | "

f"LR: {current_lr:.8f}"

)

history.append({

"epoch": epoch + 1,

"train_loss": train_loss,

"train_auc": train_metrics["auc"],

"train_acc": train_metrics["acc"],

"train_sensitivity": train_metrics["sensitivity"],

"train_specificity": train_metrics["specificity"],

"train_precision": train_metrics["precision"],

"train_f1": train_metrics["f1"],

"val_loss": val_loss,

"val_auc": val_metrics_best["auc"],

"val_acc": val_metrics_best["acc"],

"val_sensitivity": val_metrics_best["sensitivity"],

"val_specificity": val_metrics_best["specificity"],

"val_precision": val_metrics_best["precision"],

"val_f1": val_metrics_best["f1"],

"val_best_threshold": best_threshold,

"lr": current_lr,

})

if val_metrics_best["auc"] > best_auc + min_delta:

best_auc = val_metrics_best["auc"]

best_epoch = epoch + 1

best_threshold_global = best_threshold

best_model_wts = copy.deepcopy(model.state_dict())

early_stop_counter = 0

torch.save(

{

"epoch": best_epoch,

"model_state_dict": best_model_wts,

"optimizer_state_dict": optimizer.state_dict(),

"best_auc": best_auc,

"best_threshold": best_threshold_global,

"seed": Config.SEED,

"in_chans": Config.IN_CHANNELS,

},

os.path.join(output_dir, "best_model.pth"),

)

(

train_eval_loss,

train_eval_metrics_05,

train_eval_metrics,

train_best_threshold,

train_names,

train_labels,

train_probs,

train_features,

) = evaluate_and_collect(model, train_eval_loader, criterion, device)

(

val_eval_loss,

val_eval_metrics_05,

val_eval_metrics,

val_best_threshold,

val_names,

val_labels,

val_probs,

val_features,

) = evaluate_and_collect(model, val_loader, criterion, device)

save_results(train_names, train_labels, train_probs, train_features, output_dir, "best_train")

save_results(val_names, val_labels, val_probs, val_features, output_dir, "best_val")

save_metrics_csv(train_eval_metrics, output_dir, "best_train_metrics.csv")

save_metrics_csv(val_eval_metrics, output_dir, "best_val_metrics.csv")

print(

f"[*] Best model saved at epoch {best_epoch}, "

f"Val AUC={best_auc:.4f}, "

f"Val Thr={best_threshold_global:.4f}, "

f"Train AUC={train_eval_metrics['auc']:.4f}"

)

else:

early_stop_counter += 1

print(f"EarlyStopping counter: {early_stop_counter}/{Config.PATIENCE}")

if early_stop_counter >= Config.PATIENCE:

print("Early stopping triggered.")

break

history_df = pd.DataFrame(history)

history_df.to_csv(

os.path.join(output_dir, "train_log.csv"),

index=False,

encoding="utf-8-sig"

)

model.load_state_dict(best_model_wts)

(

final_train_loss,

final_train_metrics_05,

final_train_metrics,

final_train_best_threshold,

final_train_names,

final_train_labels,

final_train_probs,

final_train_features,

) = evaluate_and_collect(model, train_eval_loader, criterion, device)

(

final_val_loss,

final_val_metrics_05,

final_val_metrics,

final_val_best_threshold,

final_val_names,

final_val_labels,

final_val_probs,

final_val_features,

) = evaluate_and_collect(model, val_loader, criterion, device)

save_results(final_train_names, final_train_labels, final_train_probs, final_train_features, output_dir, "best_train")

save_results(final_val_names, final_val_labels, final_val_probs, final_val_features, output_dir, "best_val")

save_metrics_csv(final_train_metrics, output_dir, "best_train_metrics.csv")

save_metrics_csv(final_val_metrics, output_dir, "best_val_metrics.csv")

print("\n========== Training Finished ==========")

print(f"Best Epoch: {best_epoch}")

print(f"Best Val AUC: {best_auc:.4f}")

print(f"Best Val Threshold: {best_threshold_global:.4f}")

print(f"Best Train AUC: {final_train_metrics['auc']:.4f}")

print(f"训练集结果已输出到: {output_dir}")

print(f"验证集结果已输出到: {output_dir}")

if __name__ == "__main__":

main()
