# Supplementary material for: A multimodal fusion model integrating Vision Transformer, radiomics, and clinical features for predicting bone metastasis in prostate cancer
Source: Front Oncol. 2026 Jul 6;16:1841761. doi: 10.3389/fonc.2026.1841761 (PMC13381310; doi:10.3389/fonc.2026.1841761)
Supplement: Supplementary file 5 [file Table2.doc]

import os

import warnings

import numpy as np

import pandas as pd

import matplotlib.pyplot as plt

import seaborn as sns

import shap

from scipy.stats import chi2

from scipy import stats

from sklearn.linear_model import LogisticRegression

from sklearn.metrics import (

roc_curve, auc, roc_auc_score, confusion_matrix,

accuracy_score, f1_score

)

from sklearn.calibration import calibration_curve

warnings.filterwarnings("ignore")

# =========================

# 0. Global settings

# =========================

plt.rcParams["font.sans-serif"] = ["Arial", "DejaVu Sans", "Microsoft YaHei", "SimHei"]

plt.rcParams["axes.unicode_minus"] = False

RANDOM_STATE = 42

N_BOOTSTRAP = 2000

THRESHOLD = 0.5

N_BINS_CALIBRATION = 10

# =========================

# Model names / file names

# =========================

MODEL_NAME_MAP = {

"ViT模型": "Model_ViT",

"影像组学模型": "Model_Rad",

"临床模型": "Model_Clin",

"融合模型": "Model_Fusion"

}

MODEL_COLORS = {

"ViT模型": "#F0C808",

"影像组学模型": "#00A6D6",

"临床模型": "#8BC34A",

"融合模型": "#B71C1C"

}

# =========================

# 1. Path settings

# =========================

CSV_PATH = r"E:\360MoveData\Users\Home\Desktop\liguobo\pred_total_results.csv"

OUTPUT_DIR = r"./model_results"

# =========================

# 2. Column settings

# =========================

LABEL_COL = "label"

SPLIT_COL = "split"

VIT_COL = "vit_pred"

RAD_COL = "rad_pred"

CLIN_COL = "clin_pred"

TRAIN_SPLIT_NAME = "train"

VALID_SPLIT_CANDIDATES = ["val", "valid", "validation", "test"]

# =========================

# 3. Utility functions

# =========================

def ensure_dir(path):

if not os.path.exists(path):

os.makedirs(path)

def format_p_value(p):

if pd.isna(p):

return ""

if p < 0.001:

return "<0.001"

return f"{p:.3f}"

def auc_ci_bootstrap(y_true, y_score, n_bootstrap=2000, random_state=42):

rng = np.random.RandomState(random_state)

y_true = np.asarray(y_true).astype(int)

y_score = np.asarray(y_score).astype(float)

auc_val = roc_auc_score(y_true, y_score)

boot_scores = []

n = len(y_true)

for _ in range(n_bootstrap):

indices = rng.randint(0, n, n)

if len(np.unique(y_true[indices])) < 2:

continue

score = roc_auc_score(y_true[indices], y_score[indices])

boot_scores.append(score)

if len(boot_scores) == 0:

return auc_val, np.nan, np.nan

lower = np.percentile(boot_scores, 2.5)

upper = np.percentile(boot_scores, 97.5)

return auc_val, lower, upper

def hosmer_lemeshow_test(y_true, y_prob, g=10):

y_true = np.asarray(y_true).astype(int)

y_prob = np.asarray(y_prob).astype(float)

data = pd.DataFrame({"y": y_true, "p": y_prob}).copy()

try:

data["bin"] = pd.qcut(data["p"], q=g, duplicates="drop")

except Exception:

return np.nan, np.nan

obs = data.groupby("bin")["y"].agg(["sum", "count"])

obs.columns = ["observed_1", "total"]

obs["observed_0"] = obs["total"] - obs["observed_1"]

exp = data.groupby("bin")["p"].sum().to_frame("expected_1")

exp["expected_0"] = obs["total"] - exp["expected_1"]

hl_df = pd.concat([obs, exp], axis=1)

eps = 1e-8

hl_stat = (

((hl_df["observed_1"] - hl_df["expected_1"]) ** 2) / (hl_df["expected_1"] + eps) +

((hl_df["observed_0"] - hl_df["expected_0"]) ** 2) / (hl_df["expected_0"] + eps)

).sum()

df = max(len(hl_df) - 2, 1)

p_value = 1 - chi2.cdf(hl_stat, df)

return hl_stat, p_value

def calc_metrics(y_true, y_prob, threshold=0.5):

y_true = np.asarray(y_true).astype(int)

y_prob = np.asarray(y_prob).astype(float)

y_pred = (y_prob >= threshold).astype(int)

cm = confusion_matrix(y_true, y_pred, labels=[0, 1])

tn, fp, fn, tp = cm.ravel()

auc_val, auc_low, auc_high = auc_ci_bootstrap(

y_true, y_prob, n_bootstrap=N_BOOTSTRAP, random_state=RANDOM_STATE

)

sensitivity = tp / (tp + fn) if (tp + fn) > 0 else np.nan

specificity = tn / (tn + fp) if (tn + fp) > 0 else np.nan

accuracy = accuracy_score(y_true, y_pred)

ppv = tp / (tp + fp) if (tp + fp) > 0 else np.nan

npv = tn / (tn + fn) if (tn + fn) > 0 else np.nan

f1 = f1_score(y_true, y_pred, zero_division=0)

_, hl_p = hosmer_lemeshow_test(y_true, y_prob, g=10)

return {

"AUC": auc_val,

"AUC_lower": auc_low,

"AUC_upper": auc_high,

"AUC(95%CI)": f"{auc_val:.3f} ({auc_low:.3f}, {auc_high:.3f})" if pd.notna(auc_low) else f"{auc_val:.3f}",

"Sensitivity": sensitivity,

"Specificity": specificity,

"Accuracy": accuracy,

"PPV": ppv,

"NPV": npv,

"F1-score": f1,

"Hosmer-Lemeshow P value": hl_p,

"TN": tn,

"FP": fp,

"FN": fn,

"TP": tp

}

# =========================

# 4. DeLong test

# =========================

def compute_midrank(x):

x = np.asarray(x)

J = np.argsort(x)

Z = x[J]

N = len(x)

T = np.zeros(N, dtype=float)

i = 0

while i < N:

j = i

while j < N and Z[j] == Z[i]:

j += 1

T[i:j] = 0.5 * (i + j - 1) + 1

i = j

T2 = np.empty(N, dtype=float)

T2[J] = T

return T2

def fast_delong(predictions_sorted_transposed, label_1_count):

m = label_1_count

n = predictions_sorted_transposed.shape[1] - m

k = predictions_sorted_transposed.shape[0]

positive_examples = predictions_sorted_transposed[:, :m]

negative_examples = predictions_sorted_transposed[:, m:]

tx = np.empty((k, m), dtype=float)

ty = np.empty((k, n), dtype=float)

tz = np.empty((k, m + n), dtype=float)

for r in range(k):

tx[r, :] = compute_midrank(positive_examples[r, :])

ty[r, :] = compute_midrank(negative_examples[r, :])

tz[r, :] = compute_midrank(predictions_sorted_transposed[r, :])

aucs = tz[:, :m].sum(axis=1) / m / n - (m + 1.0) / (2.0 * n)

v01 = (tz[:, :m] - tx[:, :]) / n

v10 = 1.0 - (tz[:, m:] - ty[:, :]) / m

sx = np.cov(v01)

sy = np.cov(v10)

if k == 1:

sx = np.array([[sx]])

sy = np.array([[sy]])

delongcov = sx / m + sy / n

return aucs, delongcov

def calc_pvalue(aucs, sigma):

l = np.array([[1, -1]])

z = np.abs(np.diff(aucs)) / np.sqrt(np.dot(np.dot(l, sigma), l.T))[0, 0]

pvalue = 2 * (1 - stats.norm.cdf(z))

return float(pvalue)

def delong_roc_test(y_true, y_score_1, y_score_2):

y_true = np.asarray(y_true).astype(int)

y_score_1 = np.asarray(y_score_1).astype(float)

y_score_2 = np.asarray(y_score_2).astype(float)

order = np.argsort(-y_true)

y_true_sorted = y_true[order]

preds = np.vstack([y_score_1, y_score_2])[:, order]

label_1_count = int(np.sum(y_true_sorted))

if label_1_count == 0 or label_1_count == len(y_true_sorted):

return np.nan, np.nan, np.nan

aucs, delongcov = fast_delong(preds, label_1_count)

p_value = calc_pvalue(aucs, delongcov)

return float(aucs[0]), float(aucs[1]), float(p_value)

def delong_pairwise_matrix(y_true, pred_dict):

model_names = list(pred_dict.keys())

n_models = len(model_names)

pval_mat = pd.DataFrame(

np.ones((n_models, n_models)),

index=model_names,

columns=model_names

)

auc_diff_mat = pd.DataFrame(

np.zeros((n_models, n_models)),

index=model_names,

columns=model_names

)

rows = []

for i in range(n_models):

for j in range(i + 1, n_models):

m1 = model_names[i]

m2 = model_names[j]

auc1, auc2, p = delong_roc_test(y_true, pred_dict[m1], pred_dict[m2])

pval_mat.loc[m1, m2] = p

pval_mat.loc[m2, m1] = p

auc_diff_mat.loc[m1, m2] = auc1 - auc2

auc_diff_mat.loc[m2, m1] = auc2 - auc1

rows.append({

"Dataset": "",

"Model_1": MODEL_NAME_MAP.get(m1, m1),

"Model_2": MODEL_NAME_MAP.get(m2, m2),

"AUC1": auc1,

"AUC2": auc2,

"AUC Difference (AUC1-AUC2)": auc1 - auc2 if pd.notna(auc1) and pd.notna(auc2) else np.nan,

"DeLong P value": p

})

result_table = pd.DataFrame(rows)

return pval_mat, auc_diff_mat, result_table

# =========================

# 5. Plot functions

# =========================

def plot_roc_on_ax(ax, y_true, pred_dict, title):

for model_name, y_prob in pred_dict.items():

fpr, tpr, _ = roc_curve(y_true, y_prob)

roc_auc = auc(fpr, tpr)

display_name = MODEL_NAME_MAP.get(model_name, model_name)

ax.plot(

fpr, tpr,

lw=2,

color=MODEL_COLORS.get(model_name, None),

label=f"{display_name} (AUC={roc_auc:.3f})"

)

ax.plot([0, 1], [0, 1], linestyle="--", color="gray", lw=1.5)

ax.set_xlabel("1 - Specificity")

ax.set_ylabel("Sensitivity")

ax.set_title(title)

ax.legend(loc="lower right", fontsize=9)

ax.grid(False)

def plot_calibration_on_ax(ax, y_true, pred_dict, title, n_bins=10):

for model_name, y_prob in pred_dict.items():

frac_pos, mean_pred = calibration_curve(y_true, y_prob, n_bins=n_bins, strategy="quantile")

display_name = MODEL_NAME_MAP.get(model_name, model_name)

ax.plot(

mean_pred,

frac_pos,

marker="o",

lw=2,

color=MODEL_COLORS.get(model_name, None),

label=display_name

)

ax.plot([0, 1], [0, 1], linestyle="--", color="gray", label="Ideal")

ax.set_xlabel("Predicted Probability")

ax.set_ylabel("Observed Probability")

ax.set_title(title)

ax.legend(fontsize=9)

ax.grid(False)

def calculate_net_benefit_model_from_prob(thresh_group, y_true, y_prob):

y_true = np.asarray(y_true).astype(int)

y_prob = np.asarray(y_prob).astype(float)

net_benefit_model = np.array([])

for thresh in thresh_group:

if thresh <= 0 or thresh >= 1:

net_benefit_model = np.append(net_benefit_model, np.nan)

continue

y_pred = (y_prob > thresh).astype(int)

tn, fp, fn, tp = confusion_matrix(y_true, y_pred, labels=[0, 1]).ravel()

total = len(y_true)

net_benefit = (tp / total) - (fp / total) * (thresh / (1 - thresh))

net_benefit_model = np.append(net_benefit_model, net_benefit)

return net_benefit_model

def calculate_net_benefit_all(thresh_group, y_true):

y_true = np.asarray(y_true).astype(int)

net_benefit_all = np.array([])

tp = np.sum(y_true == 1)

fp = np.sum(y_true == 0)

total = len(y_true)

for thresh in thresh_group:

if thresh <= 0 or thresh >= 1:

net_benefit_all = np.append(net_benefit_all, np.nan)

continue

net_benefit = (tp / total) - (fp / total) * (thresh / (1 - thresh))

net_benefit_all = np.append(net_benefit_all, net_benefit)

return net_benefit_all

def plot_dca_on_ax(ax, thresh_group, pred_dict, y_true, model_names, set_name):

colors = ['crimson', 'blue', 'green', 'purple']

for model_name in model_names:

model_color = colors[model_names.index(model_name)]

y_prob = pred_dict[model_name]

net_benefit = calculate_net_benefit_model_from_prob(thresh_group, y_true, y_prob)

display_name = MODEL_NAME_MAP.get(model_name, model_name)

ax.plot(thresh_group, net_benefit, color=model_color, linewidth=2, label=display_name)

net_benefit_all = calculate_net_benefit_all(thresh_group, y_true)

ax.plot(thresh_group, net_benefit_all, color='black', linestyle='--', linewidth=2, label='Treat All')

ax.plot(thresh_group, np.zeros_like(thresh_group), color='gray', linestyle='-.', linewidth=2, label='Treat None')

ax.set_xlim(0, 1)

ax.set_ylim(-0.2, 0.6)

ax.set_xlabel('Threshold Probability')

ax.set_ylabel('Net Benefit')

ax.grid(False)

ax.legend(loc='upper right', fontsize=9)

ax.set_title(f'Decision Curve Analysis - {set_name}')

def plot_confusion_matrix_figure(y_true, y_prob, save_path, title, threshold=0.5):

y_true = np.asarray(y_true).astype(int)

y_pred = (np.asarray(y_prob) >= threshold).astype(int)

cm = confusion_matrix(y_true, y_pred, labels=[0, 1])

plt.figure(figsize=(5, 4))

ax = sns.heatmap(

cm,

annot=True,

fmt="d",

cmap="Blues",

cbar=False,

xticklabels=["Negative", "Positive"],

yticklabels=["Negative", "Positive"]

)

ax.set_xlabel("Predicted Label")

ax.set_ylabel("True Label")

ax.set_title(title)

plt.tight_layout()

plt.savefig(save_path, dpi=300, bbox_inches="tight")

plt.close()

def plot_delong_heatmap_on_ax(ax, pval_mat, title):

show_mat = pval_mat.copy()

show_mat.index = [MODEL_NAME_MAP.get(x, x) for x in show_mat.index]

show_mat.columns = [MODEL_NAME_MAP.get(x, x) for x in show_mat.columns]

annot_mat = show_mat.copy().astype(object)

for i in range(show_mat.shape[0]):

for j in range(show_mat.shape[1]):

if i == j:

annot_mat.iloc[i, j] = "-"

else:

annot_mat.iloc[i, j] = format_p_value(show_mat.iloc[i, j])

sns.heatmap(

show_mat,

annot=annot_mat,

fmt="",

cmap="RdYlBu_r",

vmin=0,

vmax=1,

square=True,

cbar=True,

cbar_kws={"label": "P value"},

ax=ax

)

ax.set_title(title)

def plot_fusion_shap(model, X_train, X_target, save_dir):

feature_names = ["ViT_pred", "Rad_pred", "Clin_pred"]

X_train_df = pd.DataFrame(X_train, columns=feature_names)

X_target_df = pd.DataFrame(X_target, columns=feature_names)

try:

explainer = shap.Explainer(model, X_train_df)

shap_values = explainer(X_target_df)

plt.figure()

shap.summary_plot(shap_values, X_target_df, show=False)

plt.tight_layout()

plt.savefig(os.path.join(save_dir, "Model_Fusion_SHAP_summary.png"), dpi=300, bbox_inches="tight")

plt.close()

shap.plots.bar(shap_values, show=False)

plt.tight_layout()

plt.savefig(os.path.join(save_dir, "Model_Fusion_SHAP_bar.png"), dpi=300, bbox_inches="tight")

plt.close()

except Exception as e:

print(f"SHAP plotting failed: {e}")

# =========================

# Combined figure functions

# =========================

def plot_combined_roc(split_pred_map, split_y_map, save_path):

fig, axes = plt.subplots(1, 2, figsize=(14, 6))

plot_roc_on_ax(axes[0], split_y_map["train"], split_pred_map["train"], "ROC Curve - Training Set")

plot_roc_on_ax(axes[1], split_y_map["validation"], split_pred_map["validation"], "ROC Curve - Validation Set")

plt.tight_layout()

plt.savefig(save_path, dpi=300, bbox_inches="tight")

plt.close()

def plot_combined_calibration(split_pred_map, split_y_map, save_path):

fig, axes = plt.subplots(1, 2, figsize=(14, 6))

plot_calibration_on_ax(axes[0], split_y_map["train"], split_pred_map["train"], "Calibration Curve - Training Set", n_bins=N_BINS_CALIBRATION)

plot_calibration_on_ax(axes[1], split_y_map["validation"], split_pred_map["validation"], "Calibration Curve - Validation Set", n_bins=N_BINS_CALIBRATION)

plt.tight_layout()

plt.savefig(save_path, dpi=300, bbox_inches="tight")

plt.close()

def plot_combined_dca(split_pred_map, split_y_map, save_path):

thresh_group = np.arange(0.01, 1.00, 0.01)

model_names = ["ViT模型", "影像组学模型", "临床模型", "融合模型"]

fig, axes = plt.subplots(1, 2, figsize=(16, 6))

plot_dca_on_ax(

ax=axes[0],

thresh_group=thresh_group,

pred_dict=split_pred_map["train"],

y_true=split_y_map["train"],

model_names=model_names,

set_name="Training Set"

)

plot_dca_on_ax(

ax=axes[1],

thresh_group=thresh_group,

pred_dict=split_pred_map["validation"],

y_true=split_y_map["validation"],

model_names=model_names,

set_name="Validation Set"

)

plt.tight_layout()

plt.savefig(save_path, dpi=300, bbox_inches="tight")

plt.close()

def plot_combined_delong(split_pval_map, save_path):

fig, axes = plt.subplots(1, 2, figsize=(16, 6))

plot_delong_heatmap_on_ax(axes[0], split_pval_map["train"], "DeLong Test Heatmap - Training Set")

plot_delong_heatmap_on_ax(axes[1], split_pval_map["validation"], "DeLong Test Heatmap - Validation Set")

plt.tight_layout()

plt.savefig(save_path, dpi=300, bbox_inches="tight")

plt.close()

# =========================

# 6. Train and predict

# =========================

def train_logistic_models(train_df):

y_train = train_df[LABEL_COL].values.astype(int)

model_vit = LogisticRegression(random_state=RANDOM_STATE, solver="liblinear")

model_vit.fit(train_df[[VIT_COL]], y_train)

model_rad = LogisticRegression(random_state=RANDOM_STATE, solver="liblinear")

model_rad.fit(train_df[[RAD_COL]], y_train)

model_clin = LogisticRegression(random_state=RANDOM_STATE, solver="liblinear")

model_clin.fit(train_df[[CLIN_COL]], y_train)

model_fusion = LogisticRegression(random_state=RANDOM_STATE, solver="liblinear")

model_fusion.fit(train_df[[VIT_COL, RAD_COL, CLIN_COL]], y_train)

return {

"ViT模型": model_vit,

"影像组学模型": model_rad,

"临床模型": model_clin,

"融合模型": model_fusion

}

def get_predictions(models, df):

pred_dict = {

"ViT模型": models["ViT模型"].predict_proba(df[[VIT_COL]])[:, 1],

"影像组学模型": models["影像组学模型"].predict_proba(df[[RAD_COL]])[:, 1],

"临床模型": models["临床模型"].predict_proba(df[[CLIN_COL]])[:, 1],

"融合模型": models["融合模型"].predict_proba(df[[VIT_COL, RAD_COL, CLIN_COL]])[:, 1]

}

return pred_dict

def normalize_split_name(split_name):

s = str(split_name).strip().lower()

if s == TRAIN_SPLIT_NAME.lower():

return "train"

if s in VALID_SPLIT_CANDIDATES:

return "validation"

return s

# =========================

# 7. Main

# =========================

def main():

ensure_dir(OUTPUT_DIR)

df = pd.read_csv(CSV_PATH)

df.columns = df.columns.str.strip()

print("Actual CSV columns:")

print(df.columns.tolist())

required_cols = [LABEL_COL, SPLIT_COL, VIT_COL, RAD_COL, CLIN_COL]

missing_cols = [c for c in required_cols if c not in df.columns]

if len(missing_cols) > 0:

raise ValueError(

f"Missing columns in CSV: {missing_cols}\n"

f"Current columns: {df.columns.tolist()}"

)

df = df.dropna(subset=required_cols).copy()

df[LABEL_COL] = df[LABEL_COL].astype(int)

df["split_std"] = df[SPLIT_COL].apply(normalize_split_name)

if "train" not in df["split_std"].unique():

raise ValueError("Training set was not found in split column.")

if "validation" not in df["split_std"].unique():

raise ValueError("Validation set was not found in split column. Please ensure split contains val/valid/validation/test.")

ensure_dir(os.path.join(OUTPUT_DIR, "ROC"))

ensure_dir(os.path.join(OUTPUT_DIR, "DCA"))

ensure_dir(os.path.join(OUTPUT_DIR, "Calibration"))

ensure_dir(os.path.join(OUTPUT_DIR, "ConfusionMatrix"))

ensure_dir(os.path.join(OUTPUT_DIR, "DeLong"))

ensure_dir(os.path.join(OUTPUT_DIR, "SHAP"))

ensure_dir(os.path.join(OUTPUT_DIR, "CombinedFigures"))

train_df = df[df["split_std"] == "train"].copy()

valid_df = df[df["split_std"] == "validation"].copy()

if train_df[LABEL_COL].nunique() < 2:

raise ValueError("The training set contains only one class and cannot be used for logistic regression.")

models = train_logistic_models(train_df)

plot_fusion_shap(

model=models["融合模型"],

X_train=train_df[[VIT_COL, RAD_COL, CLIN_COL]].values,

X_target=train_df[[VIT_COL, RAD_COL, CLIN_COL]].values,

save_dir=os.path.join(OUTPUT_DIR, "SHAP")

)

all_metrics_rows = []

all_delong_rows = []

split_pred_map = {}

split_y_map = {}

split_pval_map = {}

for split_std, split_display in [("train", "Training Set"), ("validation", "Validation Set")]:

split_df = df[df["split_std"] == split_std].copy()

y_true = split_df[LABEL_COL].values.astype(int)

if len(np.unique(y_true)) < 2:

print(f"Skip {split_display}: labels are not binary.")

continue

pred_dict = get_predictions(models, split_df)

split_pred_map[split_std] = pred_dict

split_y_map[split_std] = y_true

# 1. Metrics

for model_name, y_prob in pred_dict.items():

m = calc_metrics(y_true, y_prob, threshold=THRESHOLD)

all_metrics_rows.append({

"Dataset": split_display,

"Model": MODEL_NAME_MAP.get(model_name, model_name),

"AUC": round(m["AUC"], 3) if pd.notna(m["AUC"]) else np.nan,

"AUC Lower": round(m["AUC_lower"], 3) if pd.notna(m["AUC_lower"]) else np.nan,

"AUC Upper": round(m["AUC_upper"], 3) if pd.notna(m["AUC_upper"]) else np.nan,

"AUC (95% CI)": m["AUC(95%CI)"],

"Sensitivity": round(m["Sensitivity"], 3) if pd.notna(m["Sensitivity"]) else np.nan,

"Specificity": round(m["Specificity"], 3) if pd.notna(m["Specificity"]) else np.nan,

"Accuracy": round(m["Accuracy"], 3) if pd.notna(m["Accuracy"]) else np.nan,

"PPV": round(m["PPV"], 3) if pd.notna(m["PPV"]) else np.nan,

"NPV": round(m["NPV"], 3) if pd.notna(m["NPV"]) else np.nan,

"F1-score": round(m["F1-score"], 3) if pd.notna(m["F1-score"]) else np.nan,

"Hosmer-Lemeshow P value": format_p_value(m["Hosmer-Lemeshow P value"]),

"TN": m["TN"],

"FP": m["FP"],

"FN": m["FN"],

"TP": m["TP"]

})

# 2. Single confusion matrix figures

for model_name, y_prob in pred_dict.items():

file_model_name = MODEL_NAME_MAP.get(model_name, model_name).replace("/", "_")

display_model_name = MODEL_NAME_MAP.get(model_name, model_name)

plot_confusion_matrix_figure(

y_true=y_true,

y_prob=y_prob,

save_path=os.path.join(OUTPUT_DIR, "ConfusionMatrix", f"CM_{split_std}_{file_model_name}.png"),

title=f"Confusion Matrix - {split_display} - {display_model_name}",

threshold=THRESHOLD

)

# 3. DeLong

pval_mat, auc_diff_mat, delong_table = delong_pairwise_matrix(y_true, pred_dict)

delong_table["Dataset"] = split_display

all_delong_rows.append(delong_table)

split_pval_map[split_std] = pval_mat

pval_mat_save = pval_mat.copy()

pval_mat_save.index = [MODEL_NAME_MAP.get(x, x) for x in pval_mat_save.index]

pval_mat_save.columns = [MODEL_NAME_MAP.get(x, x) for x in pval_mat_save.columns]

auc_diff_mat_save = auc_diff_mat.copy()

auc_diff_mat_save.index = [MODEL_NAME_MAP.get(x, x) for x in auc_diff_mat_save.index]

auc_diff_mat_save.columns = [MODEL_NAME_MAP.get(x, x) for x in auc_diff_mat_save.columns]

pval_mat_save.to_csv(os.path.join(OUTPUT_DIR, "DeLong", f"DeLong_Pvalue_Matrix_{split_std}.csv"), encoding="utf-8-sig")

auc_diff_mat_save.to_csv(os.path.join(OUTPUT_DIR, "DeLong", f"DeLong_AUC_Difference_Matrix_{split_std}.csv"), encoding="utf-8-sig")

# Save tables

metrics_df = pd.DataFrame(all_metrics_rows)

metrics_df.to_csv(os.path.join(OUTPUT_DIR, "Model_Performance_Summary.csv"), index=False, encoding="utf-8-sig")

if len(all_delong_rows) > 0:

delong_df = pd.concat(all_delong_rows, axis=0, ignore_index=True)

delong_df["DeLong P value (formatted)"] = delong_df["DeLong P value"].apply(format_p_value)

delong_df.to_csv(os.path.join(OUTPUT_DIR, "DeLong_Pairwise_Comparison_Summary.csv"), index=False, encoding="utf-8-sig")

# Combined figures

if "train" in split_pred_map and "validation" in split_pred_map:

plot_combined_roc(

split_pred_map=split_pred_map,

split_y_map=split_y_map,

save_path=os.path.join(OUTPUT_DIR, "CombinedFigures", "ROC_Combined_Train_Validation.png")

)

plot_combined_calibration(

split_pred_map=split_pred_map,

split_y_map=split_y_map,

save_path=os.path.join(OUTPUT_DIR, "CombinedFigures", "Calibration_Combined_Train_Validation.png")

)

plot_combined_dca(

split_pred_map=split_pred_map,

split_y_map=split_y_map,

save_path=os.path.join(OUTPUT_DIR, "CombinedFigures", "DCA_Combined_Train_Validation.png")

)

plot_combined_delong(

split_pval_map=split_pval_map,

save_path=os.path.join(OUTPUT_DIR, "CombinedFigures", "DeLong_Heatmap_Combined_Train_Validation.png")

)

# Coefficients

coef_rows = []

coef_rows.append({

"Model": "Model_ViT",

"Feature": VIT_COL,

"Coefficient": models["ViT模型"].coef_[0][0],

"Intercept": models["ViT模型"].intercept_[0]

})

coef_rows.append({

"Model": "Model_Rad",

"Feature": RAD_COL,

"Coefficient": models["影像组学模型"].coef_[0][0],

"Intercept": models["影像组学模型"].intercept_[0]

})

coef_rows.append({

"Model": "Model_Clin",

"Feature": CLIN_COL,

"Coefficient": models["临床模型"].coef_[0][0],

"Intercept": models["临床模型"].intercept_[0]

})

fusion_coef = models["融合模型"].coef_[0]

fusion_intercept = models["融合模型"].intercept_[0]

for feat, coef in zip([VIT_COL, RAD_COL, CLIN_COL], fusion_coef):

coef_rows.append({

"Model": "Model_Fusion",

"Feature": feat,

"Coefficient": coef,

"Intercept": fusion_intercept

})

coef_df = pd.DataFrame(coef_rows)

coef_df.to_csv(os.path.join(OUTPUT_DIR, "Logistic_Regression_Coefficients.csv"), index=False, encoding="utf-8-sig")

print("All analyses have been completed. Results were saved to:", OUTPUT_DIR)

if __name__ == "__main__":

main()
